# Supplementary material for: Culling corallivores improves short-term coral recovery under bleaching scenarios
Source: Nat Commun. 2022 May 9;13:2520. doi: 10.1038/s41467-022-30213-x (PMC9085818; doi:10.1038/s41467-022-30213-x)
Supplement: Supplementary file 1 — Supplementary Information [file 41467_2022_30213_MOESM1_ESM.pdf]

Supplementary Information

Culling corallivores improves short-term coral recovery under bleaching scenarios

Jacob G. D. Rogers[1, 2, *] and Éva E. Plagányi[2]

[1]School of Mathematics and Physics, University of Queensland, Brisbane 4072, Queensland, Australia.

[2]CSIRO Oceans and Atmosphere, Brisbane 4072, Queensland, Australia.

[*]Corresponding author: Jacob.Rogers@csiro.au

Supplementary tables

Supplementary Table 1: Likelihood contributions arising from each data source (coral cover data and catch-per-unit-effort data (CPUE)) and encompassed penalty terms. Parameters were fitted simultaneously with each model ($A = \{0, 2.5, 5\}$) containing 68 estimated parameters. Given each model contains the same number of parameters the likelihood is used to discern relative model parsimony. Survey errors were unavailable, consequently variability was computed for each management site and is reported in brackets beside their likelihood contribution. Overall, $A = 5$ was the most likely model though this was negligibly so and all models were similar in their ability to describe the data. Model fits are illustrated in the supplementary figures. Headings within table are presented in bold.

	**Model likelihood**		
	$A = 0$	$A = 2.5$	$A = 5$
**-lnL(Total)**	-40.8655	-40.8803	-40.8865
**-lnL(Overall Coral Cover)**	-196.111	-196.131	-196.14
Management Site 1	-16.5051 (0.304919)	-16.5089 (0.30487)	-16.5118 (0.304833)
Management Site 2	-30.4181 (0.14249)	-30.4314 (0.1424)	-30.4383 (0.142353)
Management Site 3	-9.97829 (0.264074)	-9.9802 (0.264032)	-9.98091 (0.264017)
Management Site 4	-10.9938 (0.367984)	-10.9944 (0.367973)	-10.9947 (0.367969)
Management Site 5	-11.1175 (0.302751)	-11.1171 (0.302759)	-11.1169 (0.302762)
Management Site 6	-38.9448 (0.186347)	-38.9453 (0.186344)	-38.9455 (0.186343)
Management Site 7	-7.75306 (0.384404)	-7.75375 (0.384388)	-7.75438 (0.384374)
Management Site 8	-14.1783 (0.35158)	-14.1751 (0.351622)	-14.1722 (0.351662)
Management Site 9	-15.1904 (0.260824)	-15.1892 (0.260842)	-15.1883 (0.260854)
Management Site 10	-16.2836 (0.298799)	-16.2835 (0.298801)	-16.2834 (0.298802)
Management Site 11	-7.79707 (0.393305)	-7.79787 (0.393287)	-7.79821 (0.39328)
Management Site 12	-14.2116 (0.308281)	-14.2147 (0.308236)	-14.2162 (0.308214)
Management Site 13	-2.73939 (0.525094)	-2.73918 (0.5251)	-2.73908 (0.525102)
**-lnL(Overall CPUE)**	138.832	138.835	138.837
Management Site 1	9.42653 (0.884318)	9.42732 (0.884346)	9.4277 (0.884359)
Management Site 2	12.002 (1.07414)	12.0035 (1.07422)	12.0044 (1.07427)
Management Site 3	1.77279 (0.695148)	1.7703 (0.695014)	1.76908 (0.694949)
Management Site 4	11.8978 (1.04165)	11.8999 (1.04175)	11.9009 (1.0418)
Management Site 5	4.37088 (0.797064)	4.37074 (0.797057)	4.37066 (0.797053)
Management Site 6	20.1939 (1.08001)	20.1949 (1.08004)	20.1954 (1.08006)

Management Site 7	11.1224 (1.08914)	11.1225 (1.08915)	11.1226 (1.08915)
Management Site 8	18.3526 (1.14208)	18.353 (1.14209)	18.3533 (1.14211)
Management Site 9	11.736 (1.16416)	11.736 (1.16416)	11.7361 (1.16416)
Management Site 10	10.2415 (0.987768)	10.2415 (0.987768)	10.2416 (0.987768)
Management Site 11	10.5424 (1.17222)	10.5423 (1.17222)	10.5423 (1.17221)
Management Site 12	11.0751 (1.16355)	11.0751 (1.16355)	11.0751 (1.16355)
Management Site 13	6.0981 (0.868239)	6.09809 (0.868238)	6.0981 (0.868239)
Penalty terms			
Stock recruitment penalty	16.3327	16.3344	16.3356
Catch scaling penalty	0.0808	0.0813	0.0809

Supplementary Table 2: Summary of model variables and their definitions. Derivations and specification of model equations are available within the main text. Headings within table are presented in bold.

Variable	Name	Description
R_d	CoTS recruitment	Stock-recruitment relationship. The relationship is formulated in terms of slope steepness. The general relationship upon which we developed is described by Plagányi and Butterworth [1].
K^{sp}	Virgin spawning biomass (unexploited)	Spawning biomass of an unexploited population of CoTS. Computed by summing individuals within a virgin population relative R_0.
SPR_0	Spawners per recruit	Computed based on equilibrium formulation from age structure and mortality rates.
α	Stock-recruitment shape parameter	Formulated in terms of CoTS carrying capacity and spawners per recruit.
β	Stock-recruitment shape parameter	Formulated in terms of CoTS carrying capacity and spawners per recruit.
M^{CoTS}	Natural mortality of CoTS	Natural mortality is computed based on individual's age. Mortality decays to a basal level as individuals age.
a	Age	Annual age of CoTS. Value increments every 365 days.
N_a^V	Virgin population age a CoTS	Number of CoTS of a given age in unfished population. Computed based on natural mortality and equilibrium arguments.
$N_{d,a}^y$	Number of CoTS	The number of age a individuals in a given reef zone on day d during year y.
f	Coral abundance induced mortality	Scaling coefficient for the natural mortality of CoTS such that decreased preferred prey abundance leads to an increase in the starfish mortality rate. Formulated as a function of starfish increasingly switching to consumption of non-preferred coral taxa.
δ_1	Settlement impulse function	True/false function that defines the arrival of CoTS recruits. Value of 1 if argument is true, 0 otherwise.
Fp_d^y	Fished proportion of CoTS population	The proportion of all individuals within an age class at a specific location that are removed through management control.
$CPUE_d^y$	Catch-per-unit-effort	Number of CoTS injected by control diver per minute of dive time on a specific day in a given year. Catch-per-unit-effort (CPUE) acts as a proxy for population abundance upon which management control programs are based. Threshold and efficacy are communicated in terms of CPUE.
$N_{d,a}^{y,culled}$	Culled CoTS	The number of age a individuals removed on day d during year y by management actions

$C_{y,d}^{f}$	Fast-growing coral cover	Fast- growing coral in a given reef zone during year y on day d. Formulated as logistic type growth reduced by mortality sources.
$Q_{y,d}^{f}$	CoTS fast-growing coral consumption	The consumption of fast-growing coral by a population CoTS on day d of year y. Predation is fundamentally a Holling type II curve.
ρ_d	Prey switching term	Proportion of starfish predation focused on fast-growing coral on day d of year y. Computed based on the abundance of fast-growing coral prey such that, as their preferred prey abundance decreases, CoTS switch to non-preferred slow-growing corals.
$M_{y,d}^{f,Ble}$	Bleaching induced fast-growing coral mortality	Mortality inflicted upon fast-growing corals on day d of year y by a coral bleaching event. Coral mortality is calculated through the bleaching model.
$M_{y,d}^{f,Cyc}$	Cyclone induced fast-growing coral mortality	Mortality inflicted upon fast-growing corals on day d of year y by a cyclone event. Coral mortality is calculated through the cyclone model.
$C_{y,d}^{s}$	Slow-growing coral cover	Slow-growing coral in a given reef zone during year y on day d. Formulated as logistic type growth reduced by mortality sources.
$Q_{y,d}^{s}$	CoTS slow-growing coral consumption	The consumption of slow-growing coral by a population CoTS on day d of year y. Predation is fundamentally a Holling type II curve.
$M_{y,d}^{s,Ble}$	Bleaching induced slow-growing coral mortality	Mortality inflicted upon slow-growing corals on day d of year y by a coral bleaching event. Coral mortality is calculated through the bleaching model.
$M_{y,d}^{s,Cyc}$	Cyclone induced slow-growing coral mortality	Mortality inflicted upon slow-growing corals on day d of year y by a cyclone event. Coral mortality is calculated through the cyclone model.

Supplementary Table 3: Summary of parameters, their values and source/s where applicable. Prespecified denotes a parameter that is fixed prior to model fitting and tuning either due to it having a relative meaning in the model or it being collated from the literature; tuned indicates that a parameter was varied in conjunction with model fitting. Fitted parameters are presented within Supplementary Table 6. Headings within table are presented in bold and variables or parameters are presented in italics.

Parameter	Name	Value	Description	Source/s
**CoTS Beverton-Holt stock-recruitment relationship**				
R_0	Virgin recruitment	1 (Assigned)	Recruitment arising from unexploited CoTS population. Value is prespecified at 1 since the term cancels due to recursive formulation of unexploited spawning biomass.	Morello, et al. [2]
Sr	Sex ratio	0.5	Female proportion of the CoTS population.	Babcock, et al. [3]
$P_s \cdot$	Spawning participation	0.68	Proportion of reproductively mature individuals that participate in a spawning event	Babcock, et al. [4]
Fdy	Fecundity	0.5	Proportion of gonad spawned by a reproductively mature female CoTS	Rogers, et al. [5], R. C. Babcock (unpubl. data)
h_{sp}	Stock-recruitment slope steepness	0.5 (tuned)	Slope of relationship between the number of spawning adults and the number of successfully settled recruits at 20% of virgin carrying capacity.	This study
**CoTS population dynamics**				
ω	Asymptotic basal mortality	1.461 (Prespecified)	Basal mortality was fitted within the model of Plagányi, et al. [6]. Converted from an annual to daily rate when implemented in model through dividing by 365.	Plagányi, et al. [6]
λ	Age dependent decay rate	0	Controls the rate at which mortality decays to its basal rate as a function of age. Model structure is incorporated here however Morello, et al. [2] found a value of 0 most parsimonious.	Morello, et al. [2,]Plagányi, et al. [6]
a	Age	0 – 3	Annual age of CoTS. Value increments every 365 days.	This study

$\tilde{p}$	Induced coral abundance mortality	0.489	Proportion of CoTS' natural mortality that depends on the abundance of their preferred coral prey. Proportion does not need conversion to daily rate.	Plagányi, et al. [6]
$mMid$	Mortality threshold	0.1 (tuned)	A sigmoidal function is used to model threshold like behaviour of starfish mortality in response to preferred prey depletion [6]. The mortality threshold parameter is the inflection point of said sigmoidal curve.	This study
$mSlope$	Mortality threshold rate	70 (Assigned)	Controls the steepness of the threshold onset of preferred prey depletion on CoTS mortality. Parameter is selected to induce a rapid increase in mortality about the sigmoidal inflection point, $mMid$.	This study
σ_R	Recruitment variability standard deviation	0.7 (Assigned)	Controls the deterministic degree of recruitment. Stocks with greater recruitment stability have lower standard deviations, more variable stocks have higher deviations.	This study
**Management interaction with CoTS**				
ϕ_a^{CoTS}	Detectability	0.19 (age-1), 0.82 (age-2), 0.82 (age-3+)	Detectability is obtained from the fitted model of Plaganyi et al. 2020. Values relate to age-1, age-2 and age-3+ individuals respectively.	Plagányi, et al. [7,]MacNeil, et al. [8]
q	Catchability of gear/approach	0.736	Catchability is based on the fitted value of Plagányi, et al. [7]. However, data on efficacy of control relates to previous control injection technology. The catchability of the modern CoTS control program has increased so we incorporate a 10% increase in control efficacy.	This study
h	CPUE hyperstability parameter	0.5	Controls the degree of downward concavity (saturation rate) of management CPUE.	Plagányi, et al. [7]

			Parameter is fixed for model fitting purposes in Plaganyi et al. 2020.	
t_d^y	Control effort	Assigned (minutes)	Control effort is quantified in terms of dive minutes. Whilst fitting the model, recorded data for effort expended is used to fit the model. In forecasts the average effort expended at the location across all site visits is used (Supplementary Table 7).	Supplementary Table 7
**Coral dynamics**				
r^f	Intrinsic growth rate for fast-growing coral	0.50	Growth rate parameter in the logistic formulation of fast-growing coral dynamics. Annual rate of Morello et al. converted to daily rate.	Morello, et al. [2,]Plagányi, et al. [6]
K^{coral}	Joint coral carrying capacity	3000	Available space for total corals (fast- and slow-growing). Coral dynamics are formulated such that they compete for space. The overall model formulation is relative to the carrying capacity (in terms of % coral cover) and therefore the specific value of K^{coral} is arbitrary.	Morello, et al. [2]
p_2^f	Density dependent decay constant for fast growing corals	10	Controls the rate at which foraging efficiency increases with starfish density at low population abundance.	Morello, et al. [2,]Plagányi, et al. [6]
r^s	Intrinsic growth rate for slow-growing coral	0.10	Growth rate parameter in the logistic formulation of slow-growing coral dynamics. Annual rate of Morello et al. converted to daily rate.	Morello, et al. [2,]Plagányi, et al. [6]
p_1^s	Slow-growing coral consumption rate	Calculated within model	Maximum per capita consumption rate of slow-growing corals by CoTS. Consumption is based on the fast-growing coral consumption rate fitted here and the ratio of fast- to slow-growing coral consumption from Plagányi, et al. [6]. It is	Plagányi, et al. [6]

			given by p_1^f $*$(0.340/0.172). Calculation expresses consumption as a daily rate.	
p_2^s	Density dependent decay constant for slow growing corals	8	Controls the rate at which foraging efficiency increases with starfish density at low population abundance.	Morello, et al. [2,]Plagányi, et al. [6]

Supplementary Table 4: Cyclone intensity, respective frequencies, wind velocities and associated damage ranges for modelled fast and slow growing coral groups on the Great Barrier Reef. Frequency is the proportion of cyclones that fall within the respective category on the Great Barrier Reef and is based on those of Fabricius, et al. [9]. Wind velocity corresponds to the wind speeds that characterise cyclone intensities which are based on Fabricius, et al. [9] with an upper bound informed by highest observed wind speed associated with a tropical cyclone (tropical cyclone Larry, 2006) on the Great Barrier Reef since 1985 as reported by Puotinen, et al. [10]. An upper bound was necessary to sample wind speed over an interval within the model. Impulse mortality ranges for fast-growing corals, $M_{y,d}^{f,Cyc}$, and slow-growing corals, $M_{y,d}^{s,Cyc}$, resulting from a cyclone event are informed by Condie, et al. [11]. These ranges are sampled within the model for a given cyclone intensity and are proportional losses – consequent of a cyclone event – applied to pre-cyclone coral cover within the Management Site. Headings within table are presented in bold.

**Intensity**	**Frequency (%)**	**Wind velocity ($m.s^{-1}$)**	$\boldsymbol{M_{y,d}^{f,Cyc}}$ **mortality range (%)**	$\boldsymbol{M_{y,d}^{s,Cyc}}$ **mortality range (%)**
1	48	17 – 24.5	0 – 5	0
2	28	24.5 – 32.5	0 – 40	0
3	21	32.5 – 44.2	40 – 60	0 – 20
4	2	44.2 – 55.3	60 – 90	20 – 40
5	1	55.3 – 68	90 – 100	40 – 90

Supplementary Table 5: Summary of cyclone model parameters, values and their source/s. Headings within table are presented in bold and variable or parameters are presented in italics.

**Parameter**	**Meaning**	**Value**	**Source**
λ^{cyc}	Annual arrival rate of cyclone events. Converted to daily rate through division by 151.	0.4	Wolff, et al. [12]
t	Temporal resolution for cyclone rate (days)	1	This study
V	Wind speed velocity ($m.s^{-1}$) as a function of distance from cyclone centre (km)	Calculated within model	Leigh, et al. [13]
V_0	Wind velocity at gale force (17 $m.s^{-1}$)	17 $m.s^{-1}$	Fabricius, et al. [9]
V_m	Maximum wind velocity	Sampled from Supplementary Table 4	Fabricius, et al. [9], Condie, et al. [11]
d_0	Distance (km) to gale force winds from cyclone centre (cyclone radius). Based on the majority of cyclones on the GBR since 1985.	65 - 230	Puotinen, et al. [10]
d_m	Radius of maximum wind velocity (km)	Calculated within model	Leigh, et al. [13]
α	Scaling exponent for spatial velocity decay (dimensionless)	2.112458	Leigh, et al. [13]
(x_{rf}, y_{rf})	Cartesian reef centre	Sampled over model region	This study
r_1	Radius of reef (km)	0 (reefs modelled as points to limit complexity)	This study
(x_{cyc}, y_{cyc})	Cartesian tropical cyclone centre	Sampled over model region (region based on Wolff et al. 2016)	This study

Supplementary Table 6: Parameters fitted within our model across different coral thermal response scenarios alongside the spatial scale at which they were fitted. Regional scale indicates that a single parameter was estimated across all considered reefs, reef scale indicates that a single parameter was fitted for each separate reef, and sub-reef scale indicates that a single parameter was fitted for each separate management site. Parameters are presented below scoping from the regional to the sub-reef scale. Parameters were fitted simultaneously to coral cover and catch-per-unit-effort data at their respective spatial scale with standard deviations shown in brackets alongside the parameter value. The number of observations to which the model was fitted at the different scales of estimation are available in Supplementary Table 7. Headings and their description within table are presented in bold. Parameters are presented in italics.

		Value		
**Parameter**		$A = 0$	$A = 2.5$	$A = 5$
p_1^f	**Fast-growing consumption rate. Fitted at the regional scale.**			
	Reefs 1,2, 3, 4	2.29E-07 (3.89E-08)	2.29E-07 (3.89E-08)	2.29E-07 (3.89E-08)
$N_{1,1}^{2011}$	**Initial number of CoTS age-1 individuals seeded into the model on the first day of the first year (2011; 2 years prior to data period). Seeding of age-2 and age-3+ classes were based on age-1 individuals and equilibrium calculations. Fitted at the reef scale.**			
	Reef 1	175.83 (151.11)	175.18 (150.98)	174.86 (150.93)
	Reef 2	564.89 (451.05)	565.44 (451.15)	565.95 (451.19)
	Reef 3	0.45 (6.07)	0.45 (6.07)	0.45 (6.07)
	Reef 4	1114 (120.53)	1114 (120.5)	1113.9 (120.48)
$r_{y,reef}^{rec}$	**Recruitment variability parameter. Resolved at the reef scale for years 2012-2017. Fitted annually at the reef scale.**			
2012	Reef 1	0.27 (0.64)	0.27 (0.64)	0.27 (0.64)
	Reef 2	-0.11 (0.67)	-0.11 (0.67)	-0.11 (0.67)
	Reef 3	0.17 (0.71)	0.17 (0.71)	0.17 (0.71)
	Reef 4	0.05 (0.71)	0.05 (0.71)	0.05 (0.71)
2013	Reef 1	1.45 (0.41)	1.45 (0.41)	1.45 (0.41)
	Reef 2	0.74 (0.66)	0.74 (0.66)	0.74 (0.66)
	Reef 3	1.22 (0.58)	1.22 (0.58)	1.22 (0.58)
	Reef 4	-0.02 (0.65)	-0.02 (0.65)	-0.02 (0.65)
2014	Reef 1	1.15 (0.41)	1.15 (0.41)	1.15 (0.41)
	Reef 2	1.07 (0.52)	1.07 (0.52)	1.07 (0.52)
	Reef 3	0.83 (0.54)	0.83 (0.54)	0.83 (0.54)
	Reef 4	0.87 (0.65)	0.87 (0.65)	0.87 (0.65)

2015	Reef 1	-0.02 (0.47)	-0.02 (0.47)	-0.02 (0.47)
	Reef 2	-0.45 (0.56)	-0.45 (0.56)	-0.45 (0.56)
	Reef 3	-0.4 (0.54)	-0.4 (0.54)	-0.4 (0.54)
	Reef 4	0.27 (0.64)	0.27 (0.64)	0.27 (0.64)
2016	Reef 1	-1.27 (0.44)	-1.28 (0.44)	-1.28 (0.44)
	Reef 2	-0.56 (0.54)	-0.56 (0.54)	-0.56 (0.54)
	Reef 3	-0.84 (0.58)	-0.84 (0.58)	-0.84 (0.58)
	Reef 4	-0.72 (0.58)	-0.72 (0.58)	-0.72 (0.58)
2017	Reef 1	-1.76 (0.54)	-1.76 (0.54)	-1.76 (0.54)
	Reef 2	-0.61 (0.61)	-0.61 (0.61)	-0.61 (0.61)
	Reef 3	-0.85 (0.63)	-0.85 (0.63)	-0.85 (0.63)
	Reef 4	-0.45 (0.64)	-0.45 (0.64)	-0.45 (0.64)
I_d^{CoTS}	**Background CoTS immigration rates. Fitted at sub-reef scale.**			
	Management Site 1	739.04 (234.92)	739.49 (235.08)	739.7 (235.16)
	Management Site 2	649.86 (210.88)	649.96 (210.91)	649.99 (210.92)
	Management Site 3	273.64 (92.7)	273.82 (92.78)	273.91 (92.82)
	Management Site 4	559.92 (186.14)	560.32 (186.3)	560.51 (186.37)
	Management Site 5	2533.6 (796.98)	2535 (797.48)	2535.7 (797.73)
	Management Site 6	5652.5 (1651.2)	5656.2 (1652.5)	5658 (1653.1)
	Management Site 7	435.19 (159.7)	435.01 (159.63)	434.84 (159.56)
	Management Site 8	1002.7 (327.29)	1002.2 (327.12)	1001.8 (326.98)
	Management Site 9	201.45 (67.05)	201.45 (67.05)	201.45 (67.05)
	Management Site 10	371.97 (123.91)	371.98 (123.91)	371.98 (123.91)
	Management Site 11	48.16 (24.55)	48.16 (24.55)	48.16 (24.55)
	Management Site 12	357.37 (131.9)	357.36 (131.89)	357.36 (131.89)
	Management Site 13	124.2 (48.37)	124.2 (48.37)	124.2 (48.37)
DHW	**Effective Degree Heating Weeks experienced by corals during the back-to-back bleaching events of 2016-2017 on the Great Barrier Reef. Fitted at the sub-reef scale.**			
2016	Management Site 1	4.64 (0.45)	4.64 (0.45)	4.64 (0.45)
	Management Site 2	1.8 (4.39)	1.98 (3.66)	2.06 (3.41)
	Management Site 3	4.95 (0.3)	4.95 (0.3)	4.95 (0.3)
	Management Site 4	0.01 (0.05)	0.01 (0.05)	0.01 (0.05)

	Management Site 5	0.01 (0.04)	0.01 (0.04)	0.01 (0.04)
	Management Site 6	0.01 (0.25)	0.01 (0.27)	0.01 (0.27)
	Management Site 7	5.27 (0.36)	5.27 (0.36)	5.27 (0.36)
	Management Site 8	4.9 (0.37)	4.9 (0.37)	4.9 (0.37)
	Management Site 9	4.4 (0.55)	4.4 (0.55)	4.4 (0.55)
	Management Site 10	4.37 (0.66)	4.37 (0.66)	4.37 (0.66)
	Management Site 11	0.01 (0.04)	0.01 (0.05)	0.01 (0.05)
	Management Site 12	3.23 (2)	3.24 (1.97)	3.25 (1.96)
	Management Site 13	0.01 (0.07)	0.01 (0.07)	0.01 (0.07)
2017	Management Site 1	5.37 (0.31)	5.85 (0.23)	6.12 (0.28)
	Management Site 2	6.46 (0.07)	6.49 (0.1)	6.51 (0.15)
	Management Site 3	0.01 (0.07)	0.01 (0.13)	0.01 (0.19)
	Management Site 4	6.33 (0.18)	6.33 (0.18)	6.34 (0.18)
	Management Site 5	6.06 (0.17)	6.06 (0.17)	6.07 (0.17)
	Management Site 6	3.84 (0.65)	3.84 (0.65)	3.85 (0.65)
	Management Site 7	5.45 (0.37)	6.32 (0.27)	6.9 (0.38)
	Management Site 8	5.63 (0.25)	6.25 (0.19)	6.62 (0.29)
	Management Site 9	5.57 (0.22)	5.94 (0.17)	6.15 (0.24)
	Management Site 10	4.43 (0.76)	4.79 (0.61)	4.99 (0.55)
	Management Site 11	6.49 (0.12)	6.49 (0.12)	6.49 (0.12)
	Management Site 12	5.8 (0.2)	5.92 (0.17)	5.97 (0.25)
	Management Site 13	6.14 (0.18)	6.14 (0.18)	6.14 (0.18)

Supplementary Table 7: Summary of data observations and effort distribution (dive minutes). Average effort at the reef and regional scale was computed based on data entries and not average effort at the sub-reef scale top avoid mis-specifying effort at these scales. Data set is a subset of four reefs from the data set described by Westcott, et al. [14]. Headings within table are presented in bold.

Reef	Management Site	CPUE observations	Coral cover observations	Total observations	Average effort (minutes)
**Management site totals**					
1	1	25	24	49	478
1	2	21	21	42	480
1	3	13	12	25	514
1	4	22	22	44	621
1	5	16	16	32	628
1	6	35	33	68	1010
2	7	19	17	36	460
2	8	29	26	55	634
3	9	18	18	36	572
3	10	21	23	44	530
4	11	16	18	34	440
4	12	17	21	38	467
4	13	17	19	36	430
**Reef totals**					
1		132	128	260	665
2		48	43	91	565
3		39	41	80	549
4		50	58	108	446
**Overall totals**		**269**	**270**	**539**	**590**

Supplementary figures

- Supplementary figures 1-37 relate to coral cover catch-per-unit-effort rates (CPUE) for each management site. These are as per main figures Fig. 2-3 for other sites and scenarios. Specifically, they are compendium plots encompassing the model fits, thermal stress scenarios (Degree Heating Weeks; $\mathrm{DHW} \in \{\, 4, 7 \,\}$) and management scenario (no manual control, monthly manual control) for each value of coral adaptive capacity ($A \in \{0, 2.5, 5\}$).
- Supplementary figures 38-40 relate to coral cover and summarise the difference in coral cover induced by management for each $A \in \{0, 2.5, 5\}$. Differences for each Management Site are plotted alongside the mean and median across all sites under each thermal stress scenario ($\mathrm{DHW} \in \{0, 4, 7, 10\,\}$).
- Supplementary figures 41-43 relate to CPUE and summarise the difference in CPUE induced by management for each $A \in \{0, 2.5, 5\}$. Differences for each Management Site are plotted alongside the mean and median across all sites under each thermal stress scenario ($\mathrm{DHW} \in \{0, 4, 7, 10\,\}$).
- Supplementary figures 44-45 provide annual composite of maximum accumulated thermal stress maps for thermal stress events in 2016 and 2017 appended with approximate reef locations.

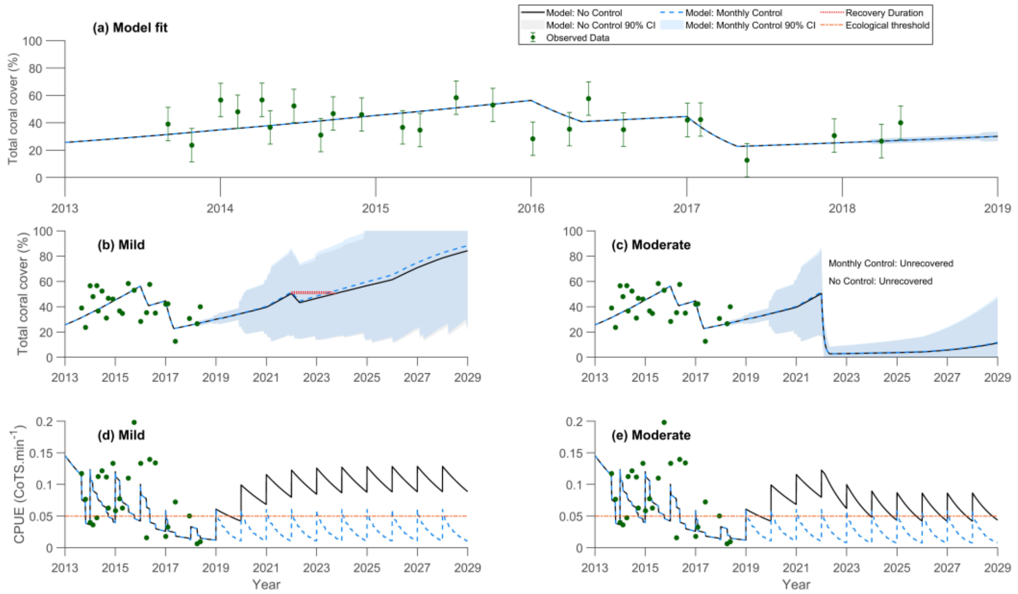

Supplementary Fig. 1: Management site 1 model fits and total coral cover (%) and catch-per-unit-effort (CPUE; CoTS.min^{-1}) trajectories under no manual control vs monthly manual control given no adaptive capacity ($A = 0$): In all panels, black solid lines define the no control scenario and the blue dashed line defines the controlled scenario. **a** The model fit to total coral cover for the management site (data points plotted with error bars depicting ± 90 % CI; green points and error bars). Each data point constitutes a single observation. Variance for the site was calculated at the Maximum Likelihood Estimate (MLE) which was simultaneously fitted to n=539 observations (both coral cover and CoTS CPUE, see Supplementary Table 7). The 90 % CIs were calculated based on the variance obtained for Management site's coral cover series and the number of observations in the series is given in Supplementary Table 7 column "Coral cover observations" at the management site level. **b-c** Total coral cover trajectories under different thermal stress levels expressed as Degree Heating Weeks ($\mathrm{DHW} \in \{4, 7\}$) simulated in year 2022. Mean trajectories are presented ± 90 % CI depicted as errors bands (n=80 simulations; grey shading is a 90 % CI error bands for the no control scenario, blue shading is 90 % CI for the controlled scenario). If coral cover recovery to pre-perturbation levels is not observed by year 2029 the management scenario is denoted, 'Unrecovered'. A lack of perturbation-induced mortality is denoted by, 'Recovery not applicable'. **d-e** CPUE trajectories under $\mathrm{DHW} = 4$ and $\mathrm{DHW} = 7$ events. CPUE is a management-based measure of CoTS abundance conditional on demographics and detectability. Error bars are not displayed in **b-e** to simplify display. Variability in each management scenario's trajectory due to stochastic tropical cyclones impacts over years 2018 – 2029 is indicated by shaded uncertainty bands and was limited in CPUE trajectories. The ecological threshold of 0.05 CPUE above which CoTS consumption exceeds coral growth based on a coral cover of 35% is plotted [7] (orange dash-dot line). CPUE sawtooth curve patterns are due to model population dynamics as individuals annually become detectable to the management program.

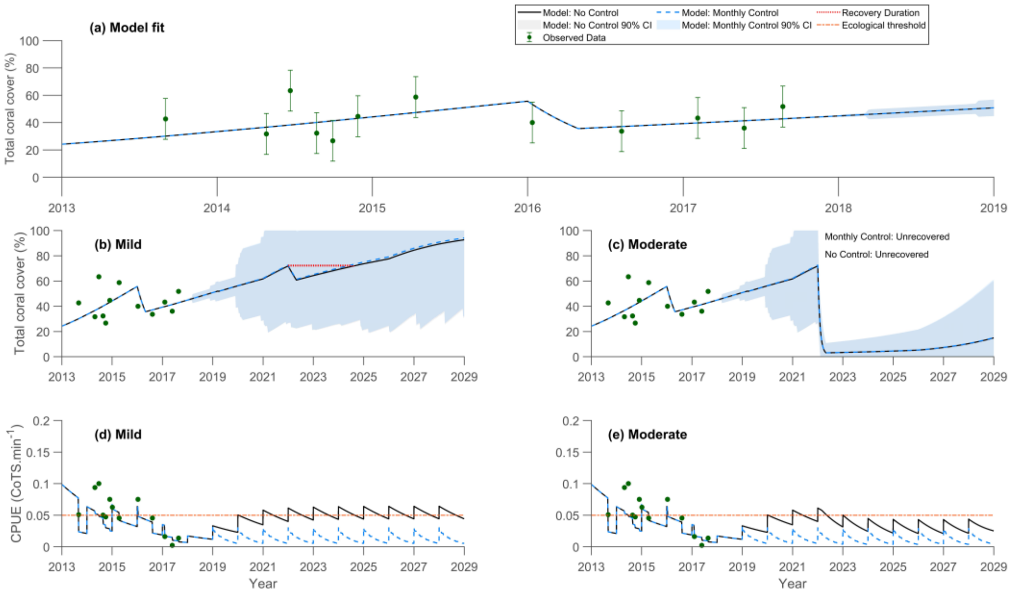

Supplementary Fig. 2: Compendium plot for Management Site 3 with details as specified in Supplementary Fig. 1. a The model fit for the management site, **b-c** total coral cover trajectories under different thermal stress levels expressed as Degree Heating Weeks ($DHW \in \{4, 7\}$) simulated in year 2022, and **d-e** Catch-per-unit-effort (CPUE) trajectories under $DHW = 4$ and $DHW = 7$ events.

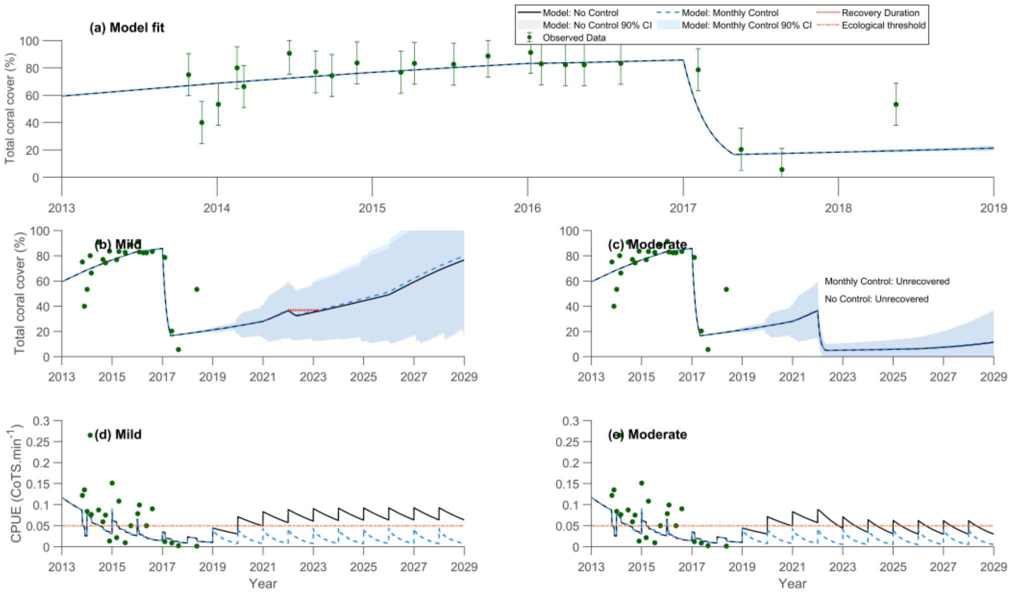

Supplementary Fig. 3: Compendium plot for Management Site 4 with details as specified in Supplementary Fig. 1. a The model fit for the management site, **b-c** total coral cover trajectories under different thermal stress levels expressed as Degree Heating Weeks ($DHW \in \{4, 7\}$) simulated in year 2022, and **d-e** Catch-per-unit-effort (CPUE) trajectories under $DHW = 4$ and $DHW = 7$ events.

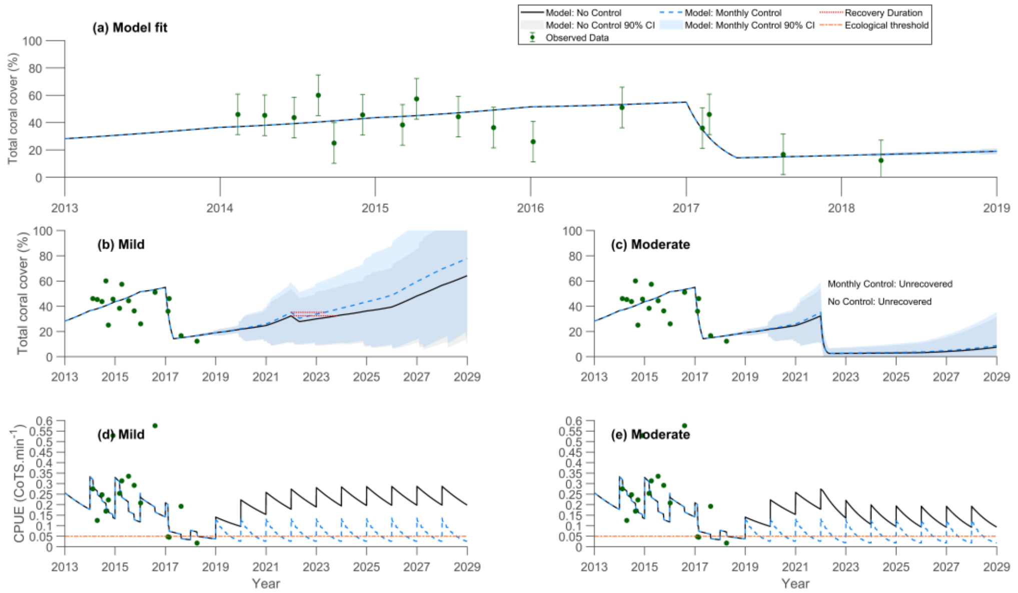

Supplementary Fig. 4: Compendium plot for Management Site 5 with details as specified in Supplementary Fig 1. a The model fit for the management site, **b-c** total coral cover trajectories under different thermal stress levels expressed as Degree Heating Weeks ($DHW \in \{4, 7\}$) simulated in year 2022, and **d-e** Catch-per-unit-effort (CPUE) trajectories under $DHW = 4$ and $DHW = 7$ events.

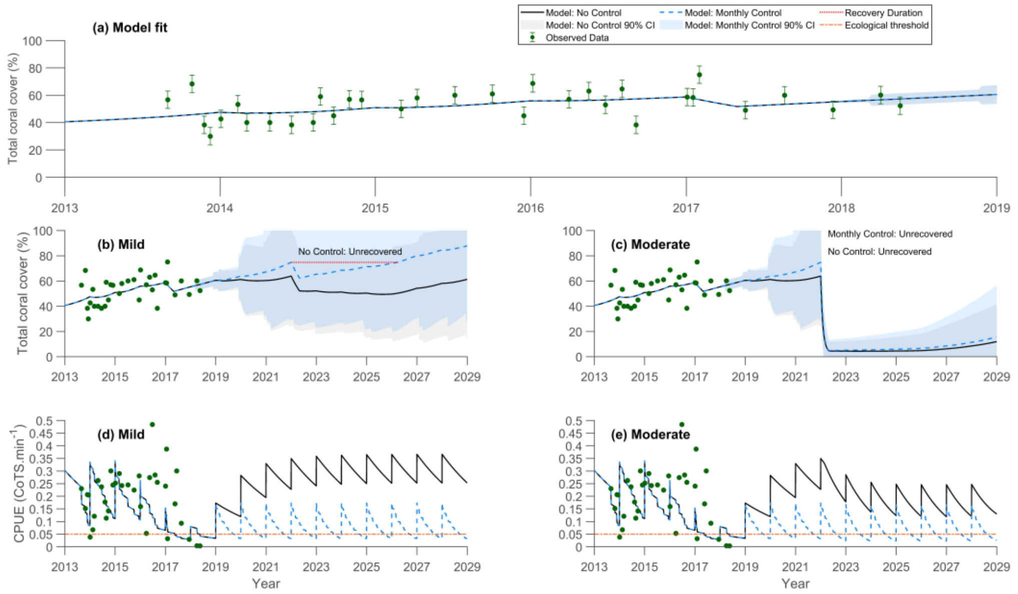

Supplementary Fig. 5: Compendium plot for Management Site 6 with details as specified in Supplementary Fig. 1. a The model fit for the management site, **b-c** total coral cover trajectories under different thermal stress levels expressed as Degree Heating Weeks ($DHW \in \{4, 7\}$) simulated in year 2022, and **d-e** Catch-per-unit-effort (CPUE) trajectories under $DHW = 4$ and $DHW = 7$ events.

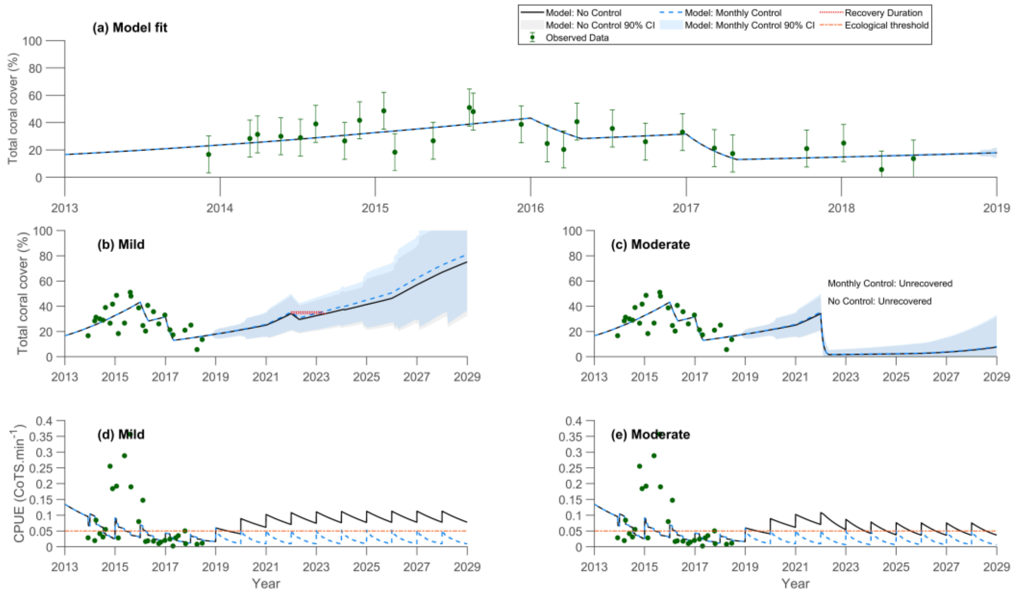

Supplementary Fig. 6: Compendium plot for Management Site 8 with details as specified in Supplementary Fig. 1. a The model fit for the management site, **b-c** total coral cover trajectories under different thermal stress levels expressed as Degree Heating Weeks ($DHW \in \{4, 7\}$) simulated in year 2022, and **d-e** Catch-per-unit-effort (CPUE) trajectories under $DHW = 4$ and $DHW = 7$ events.

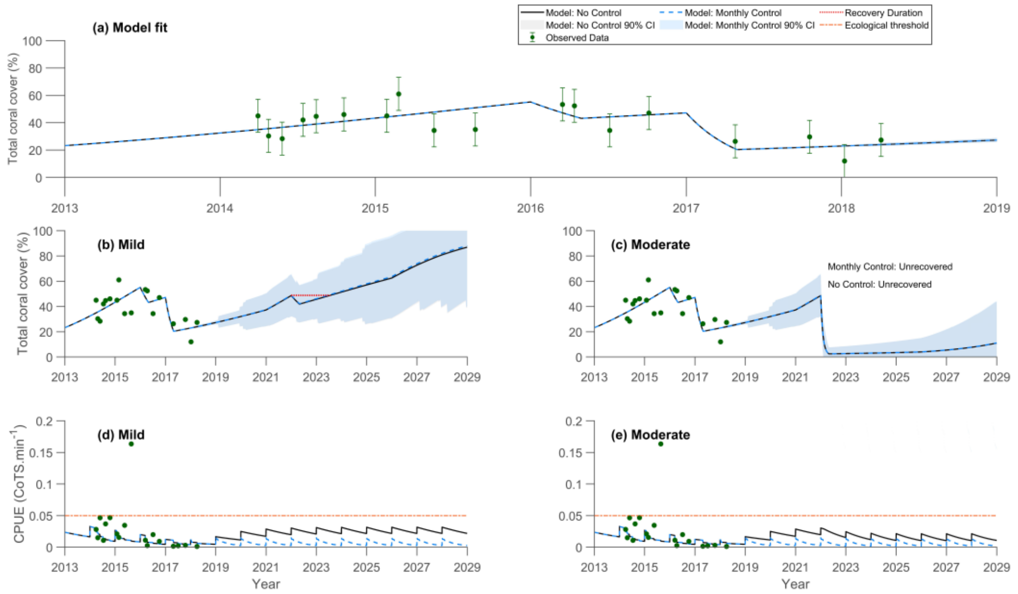

Supplementary Fig. 7: Compendium plot for Management Site 9 with details as specified in Supplementary Fig. 1. a The model fit for the management site, **b-c** total coral cover trajectories under different thermal stress levels expressed as Degree Heating Weeks ($DHW \in \{4, 7\}$) simulated in year 2022, and **d-e** Catch-per-unit-effort (CPUE) trajectories under $DHW = 4$ and $DHW = 7$ events.

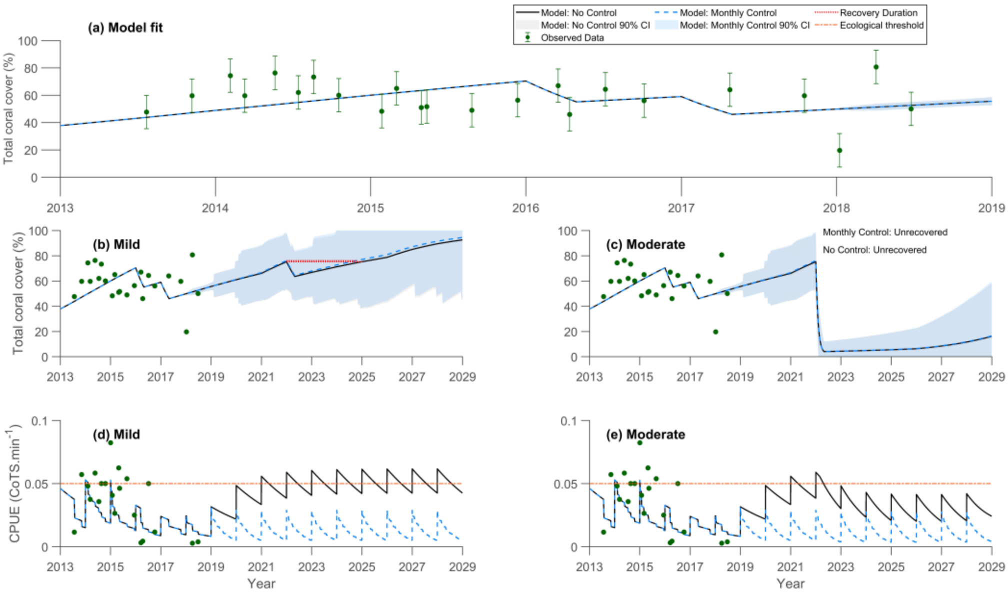

Supplementary Fig. 8: Compendium plot for Management Site 10 with details as specified in Supplementary Fig. 1. a The model fit for the management site, **b-c** total coral cover trajectories under different thermal stress levels expressed as Degree Heating Weeks ($DHW \in \{4, 7\}$) simulated in year 2022, and **d-e** Catch-per-unit-effort (CPUE) trajectories under $DHW = 4$ and $DHW = 7$ events.

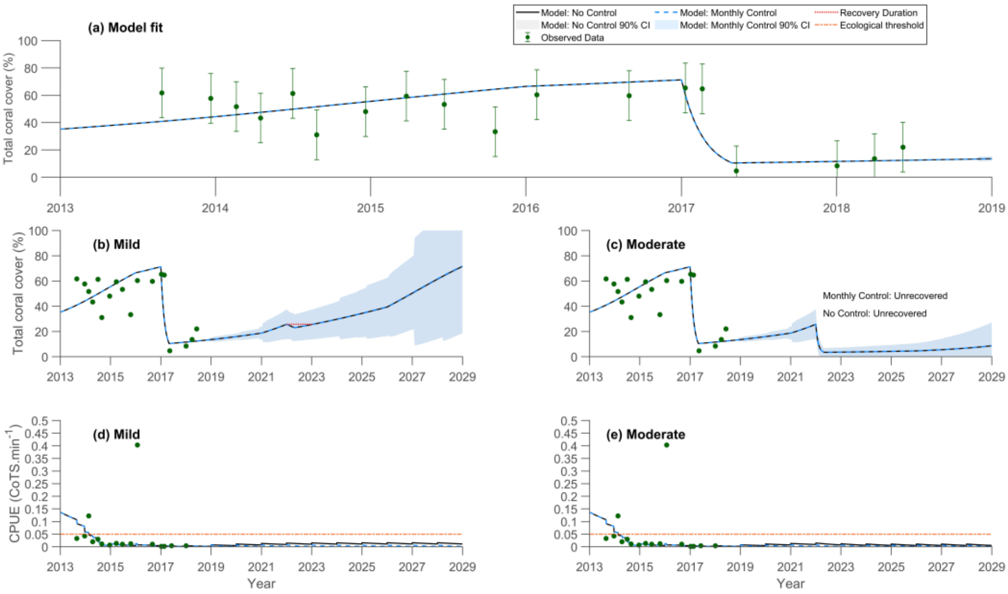

Supplementary Fig. 9: Compendium plot for Management Site 11 with details as specified in Supplementary Fig. 1. a The model fit for the management site, **b-c** total coral cover trajectories under different thermal stress levels expressed as Degree Heating Weeks ($DHW \in \{4, 7\}$) simulated in year 2022, and **d-e** Catch-per-unit-effort (CPUE) trajectories under $DHW = 4$ and $DHW = 7$ events.

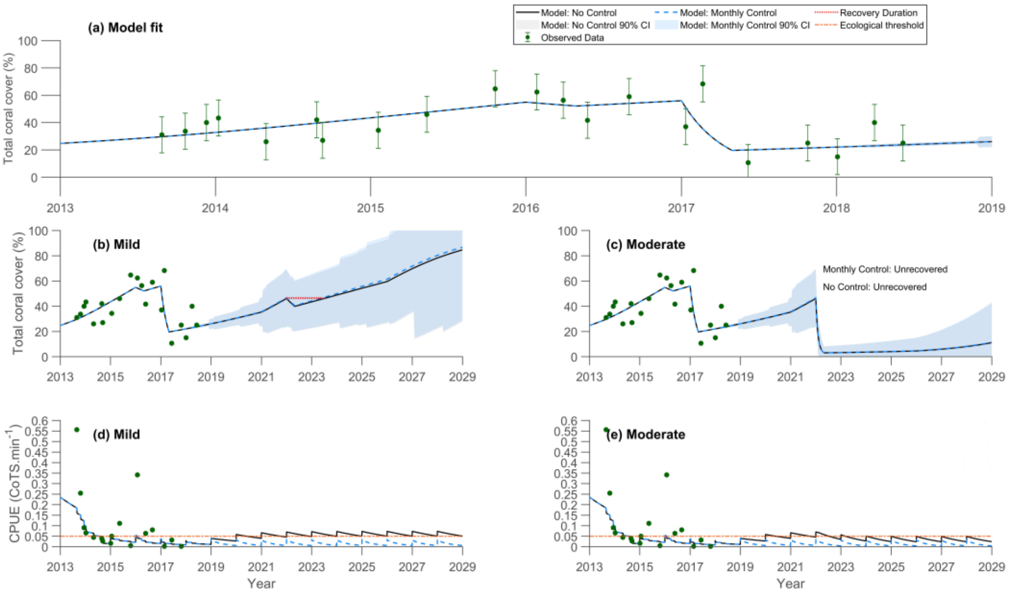

Supplementary Fig. 10: Compendium plot for Management Site 12 with details as specified in Supplementary Fig. 1. a The model fit for the management site, **b-c** total coral cover trajectories under different thermal stress levels expressed as Degree Heating Weeks ($DHW \in \{4, 7\}$) simulated in year 2022, and **d-e** Catch-per-unit-effort (CPUE) trajectories under $DHW = 4$ and $DHW = 7$ events.

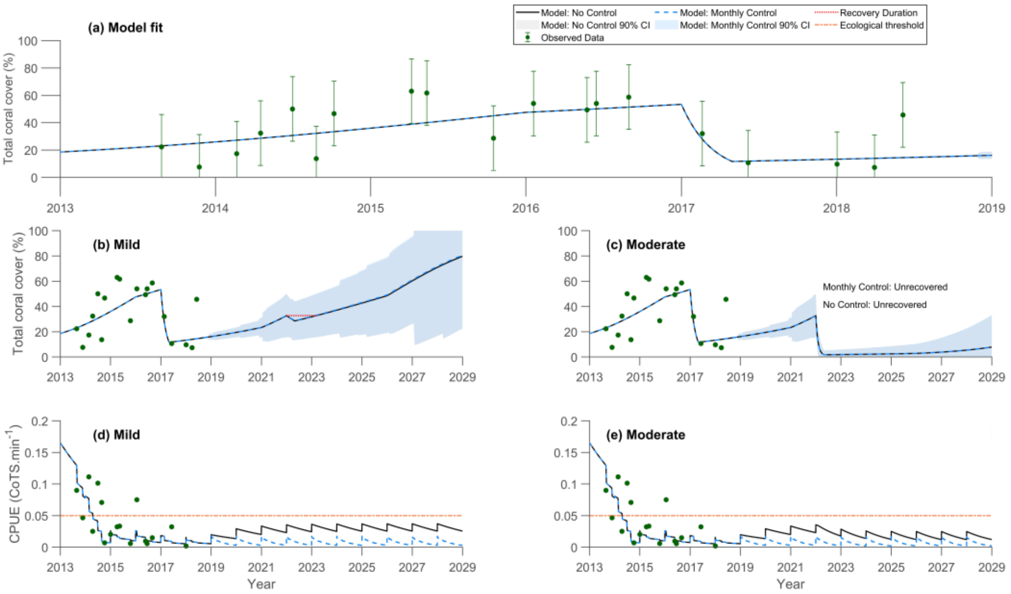

Supplementary Fig. 11: Compendium plot for Management Site 13 with details as specified in Supplementary Fig. 1. a The model fit for the management site, **b-c** total coral cover trajectories under different thermal stress levels expressed as Degree Heating Weeks ($DHW \in \{4, 7\}$) simulated in year 2022, and **d-e** Catch-per-unit-effort (CPUE) trajectories under $DHW = 4$ and $DHW = 7$ events.

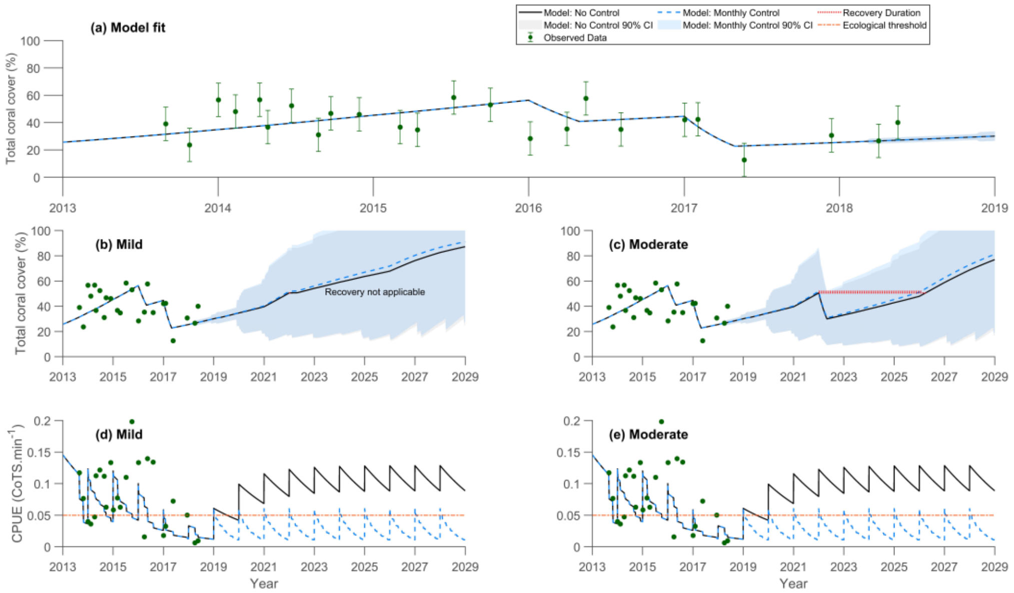

Supplementary Fig. 12: Compendium plot for Management Site 1 with details as specified in Supplementary Fig. 1 but $A = 2.5$. a The model fit for the management site, **b-c** total coral cover trajectories under different thermal stress levels expressed as Degree Heating Weeks ($DHW \in \{4, 7\}$) simulated in year 2022, and **d-e** Catch-per-unit-effort (CPUE) trajectories under $DHW = 4$ and $DHW = 7$ events.

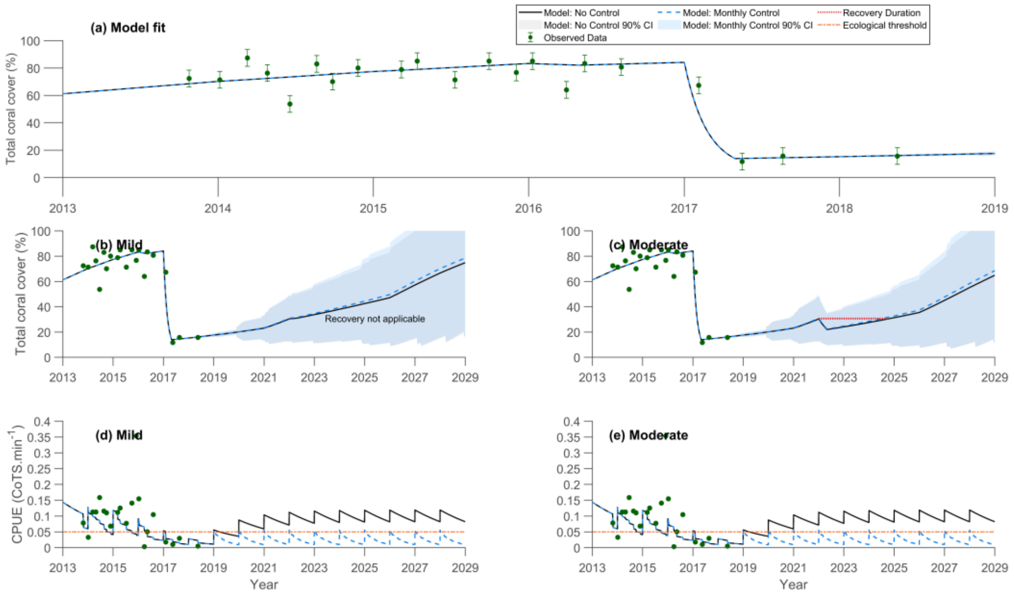

Supplementary Fig. 13: Compendium plot for Management Site 2 with details as specified in Supplementary Fig. 1 but $A = 2.5$. a The model fit for the management site, **b-c** total coral cover trajectories under different thermal stress levels expressed as Degree Heating Weeks ($DHW \in \{4, 7\}$) simulated in year 2022, and **d-e** Catch-per-unit-effort (CPUE) trajectories under $DHW = 4$ and $DHW = 7$ events.

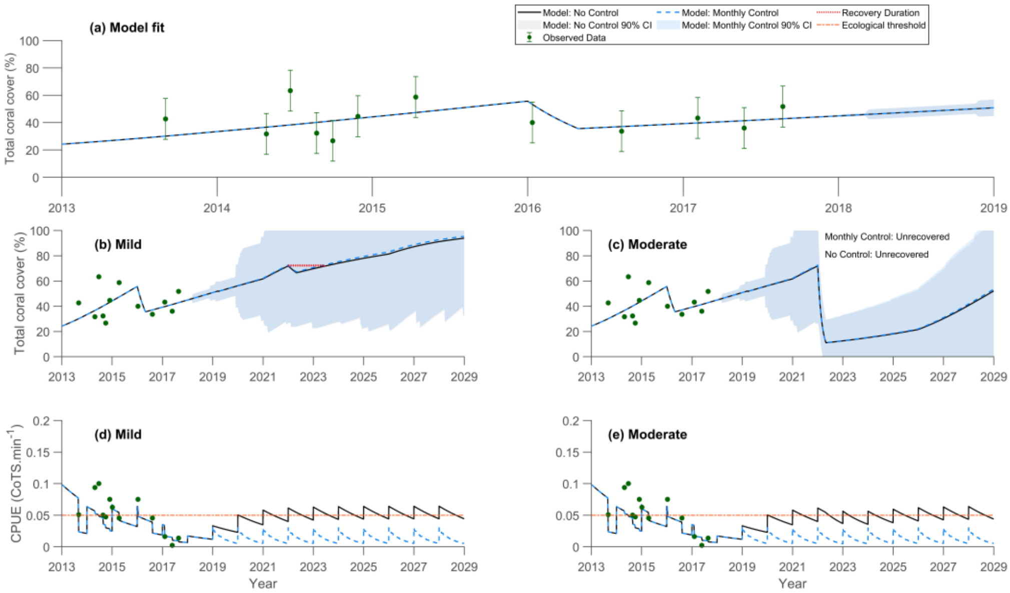

Supplementary Fig. 14: Compendium plot for Management Site 3 with details as specified in Supplementary Fig. 1 but $A = 2.5$. a The model fit for the management site, **b-c** total coral cover trajectories under different thermal stress levels expressed as Degree Heating Weeks ($DHW \in \{4, 7\}$) simulated in year 2022, and **d-e** Catch-per-unit-effort (CPUE) trajectories under $DHW = 4$ and $DHW = 7$ events.

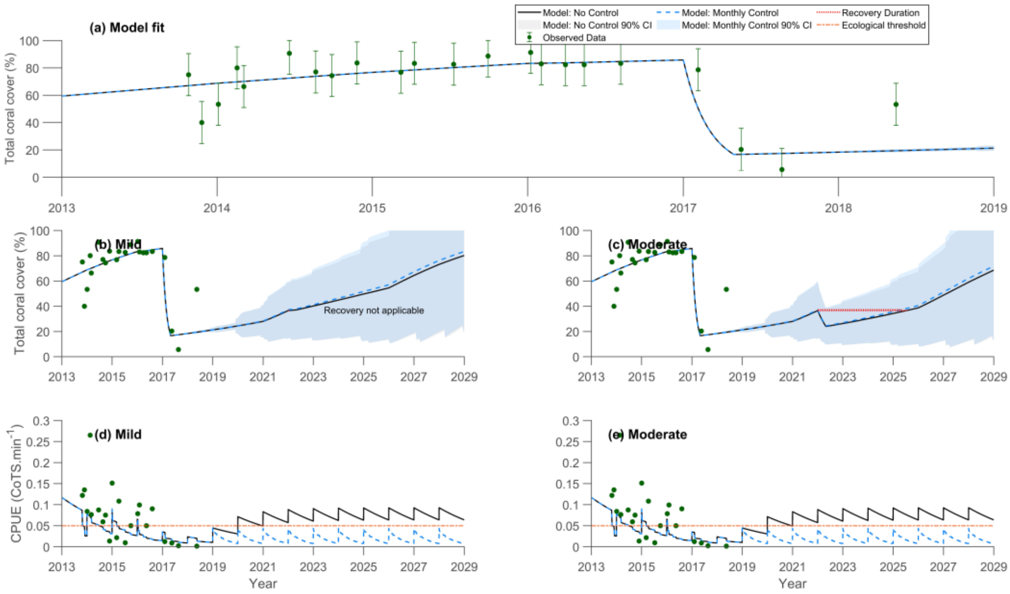

Supplementary Fig. 15: Compendium plot for Management Site 4 with details as specified in Supplementary Fig. 1 but $A = 2.5$. a The model fit for the management site, **b-c** total coral cover trajectories under different thermal stress levels expressed as Degree Heating Weeks ($DHW \in \{4, 7\}$) simulated in year 2022, and **d-e** Catch-per-unit-effort (CPUE) trajectories under $DHW = 4$ and $DHW = 7$ events.

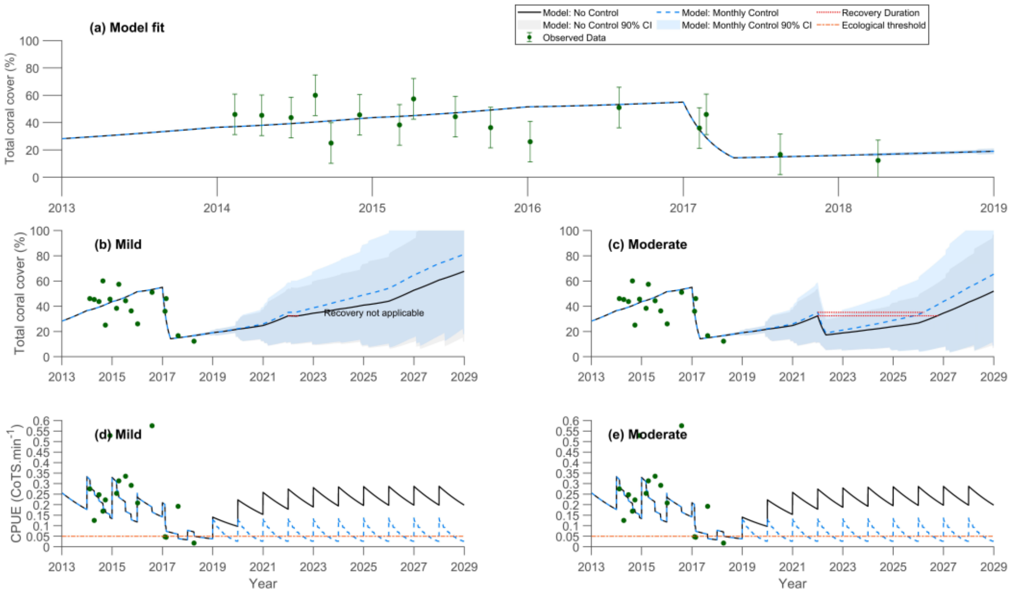

Supplementary Fig. 16: Compendium plot for Management Site 5 with details as specified in Supplementary Fig. 1 but $A = 2.5$. a The model fit for the management site, **b-c** total coral cover trajectories under different thermal stress levels expressed as Degree Heating Weeks ($DHW \in \{4, 7\}$) simulated in year 2022, and **d-e** Catch-per-unit-effort (CPUE) trajectories under $DHW = 4$ and $DHW = 7$ events.

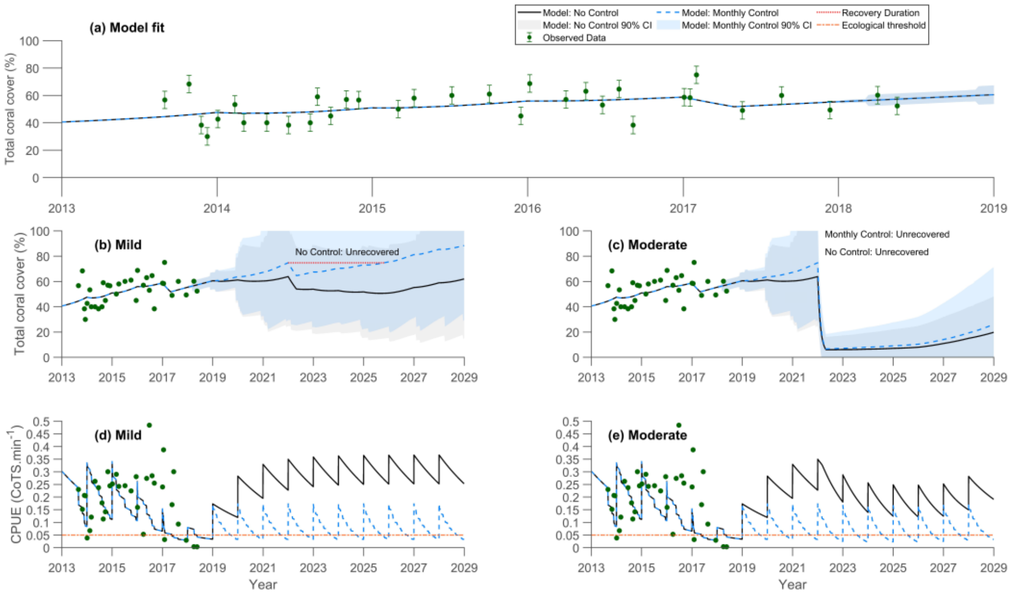

Supplementary Fig. 17: Compendium plot for Management Site 6 with details as specified in Supplementary Fig. 1 but $A = 2.5$. a The model fit for the management site, **b-c** total coral cover trajectories under different thermal stress levels expressed as Degree Heating Weeks ($DHW \in \{4, 7\}$) simulated in year 2022, and **d-e** Catch-per-unit-effort (CPUE) trajectories under $DHW = 4$ and $DHW = 7$ events.

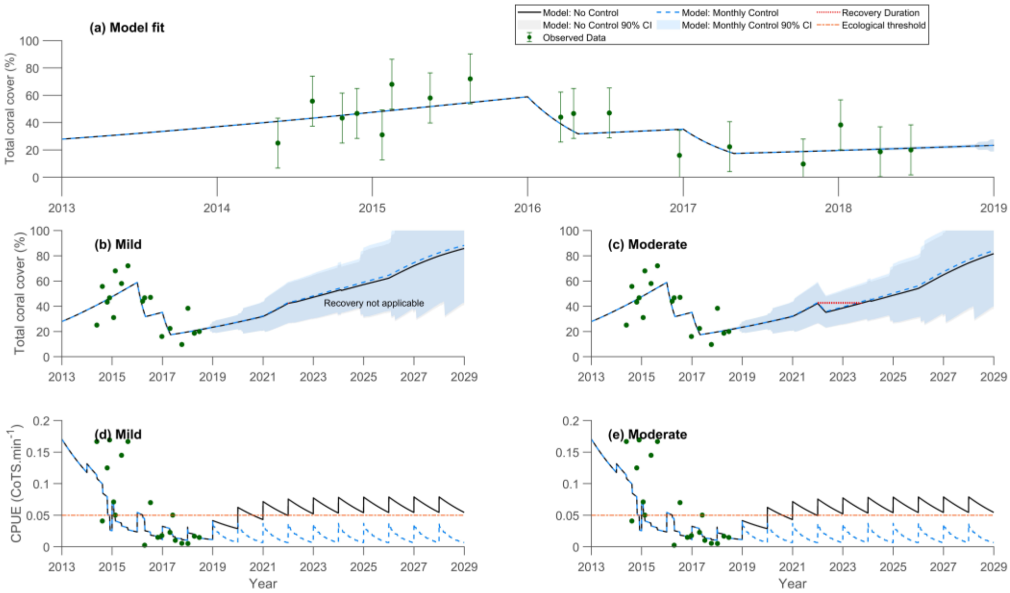

Supplementary Fig. 18: Compendium plot for Management Site 7 with details as specified in Supplementary Fig. 1 but $A = 2.5$. a The model fit for the management site, **b-c** total coral cover trajectories under different thermal stress levels expressed as Degree Heating Weeks ($DHW \in \{4, 7\}$) simulated in year 2022, and **d-e** Catch-per-unit-effort (CPUE) trajectories under $DHW = 4$ and $DHW = 7$ events.

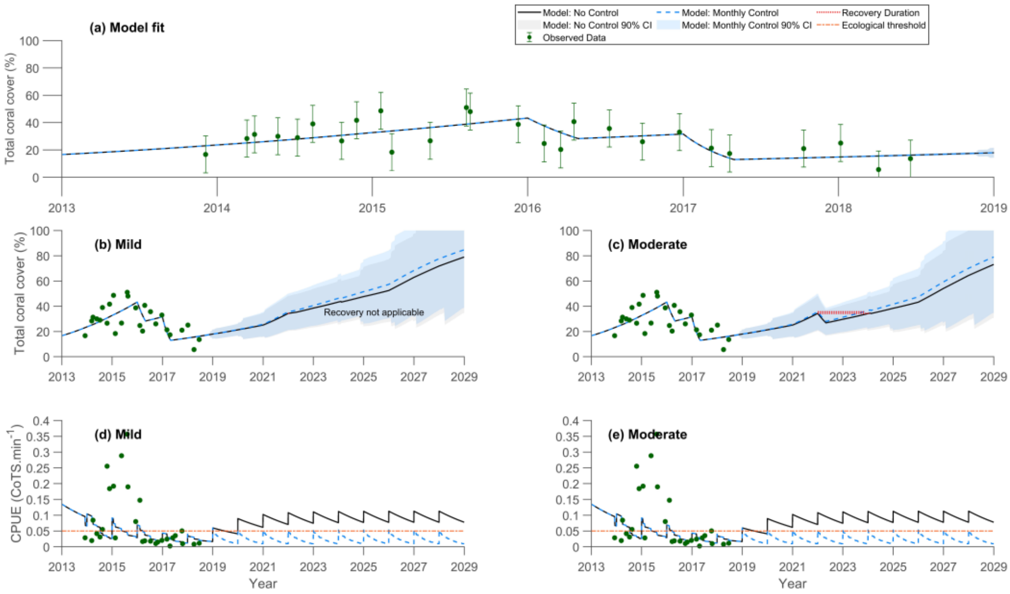

Supplementary Fig. 19: Compendium plot for Management Site 8 with details as specified in Supplementary Fig. 1 but $A = 2.5$. a The model fit for the management site, **b-c** total coral cover trajectories under different thermal stress levels expressed as Degree Heating Weeks ($DHW \in \{4, 7\}$) simulated in year 2022, and **d-e** Catch-per-unit-effort (CPUE) trajectories under $DHW = 4$ and $DHW = 7$ events.

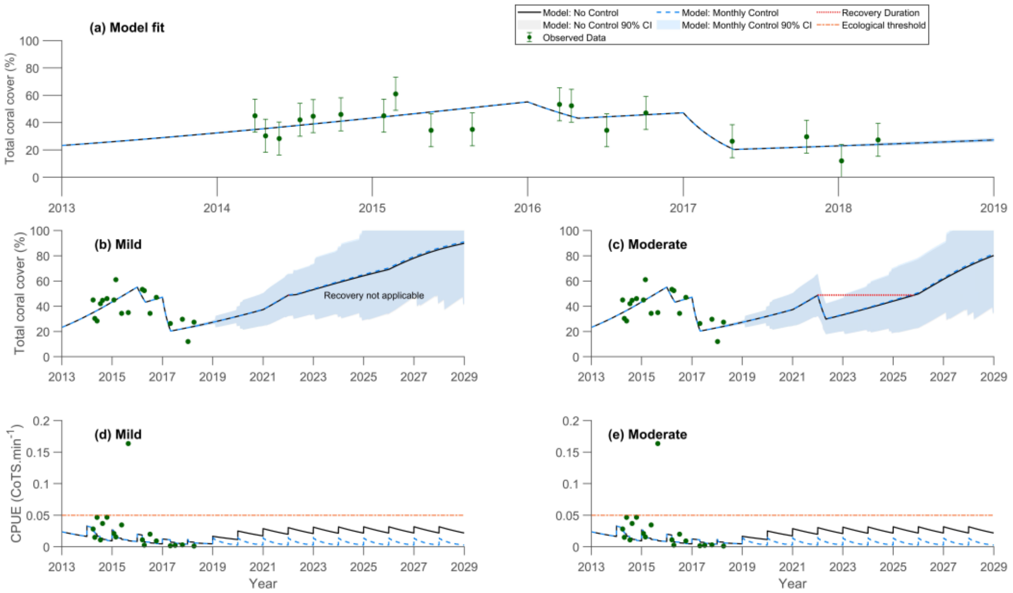

Supplementary Fig. 20: Compendium plot for Management Site 9 with details as specified in Supplementary Fig. 1 but $A = 2.5$. a The model fit for the management site, **b-c** total coral cover trajectories under different thermal stress levels expressed as Degree Heating Weeks ($DHW \in \{4, 7\}$) simulated in year 2022, and **d-e** Catch-per-unit-effort (CPUE) trajectories under $DHW = 4$ and $DHW = 7$ events.

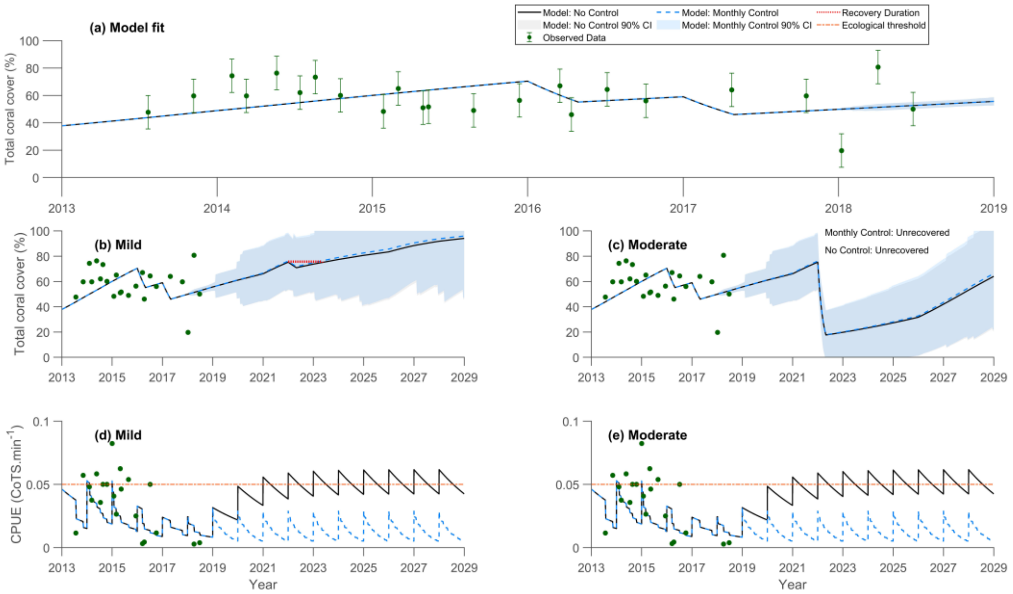

Supplementary Fig. 21: Compendium plot for Management Site 10 with details as specified in Supplementary Fig. 1 but $A = 2.5$. a The model fit for the management site, **b-c** total coral cover trajectories under different thermal stress levels expressed as Degree Heating Weeks ($DHW \in \{4, 7\}$) simulated in year 2022, and **d-e** Catch-per-unit-effort (CPUE) trajectories under $DHW = 4$ and $DHW = 7$ events.

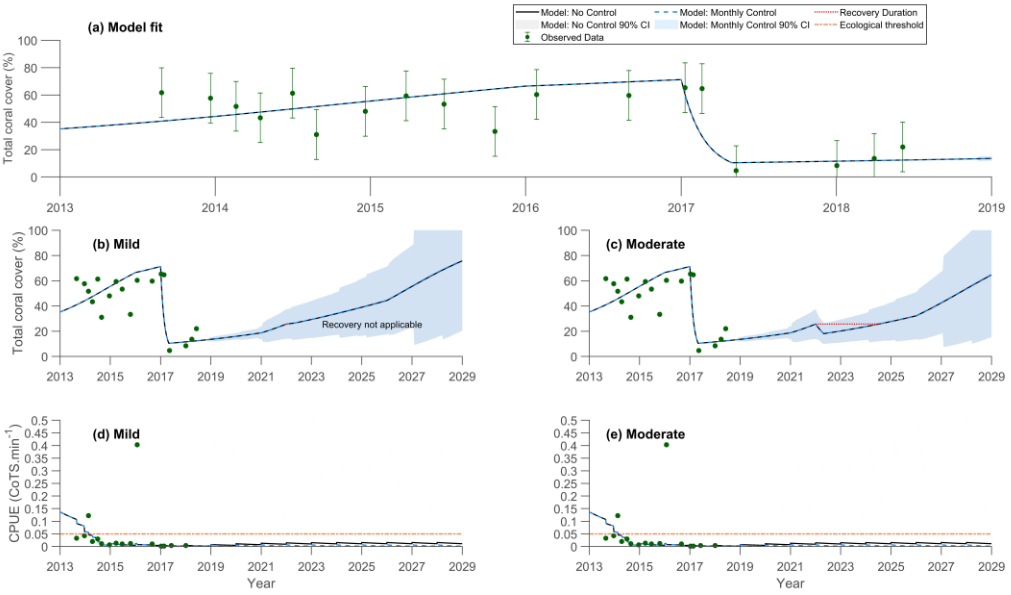

Supplementary Fig. 22: Compendium plot for Management Site 11 with details as specified in Supplementary Fig. 1 but $A = 2.5$. a The model fit for the management site, **b-c** total coral cover trajectories under different thermal stress levels expressed as Degree Heating Weeks ($DHW \in \{4, 7\}$) simulated in year 2022, and **d-e** Catch-per-unit-effort (CPUE) trajectories under $DHW = 4$ and $DHW = 7$ events.

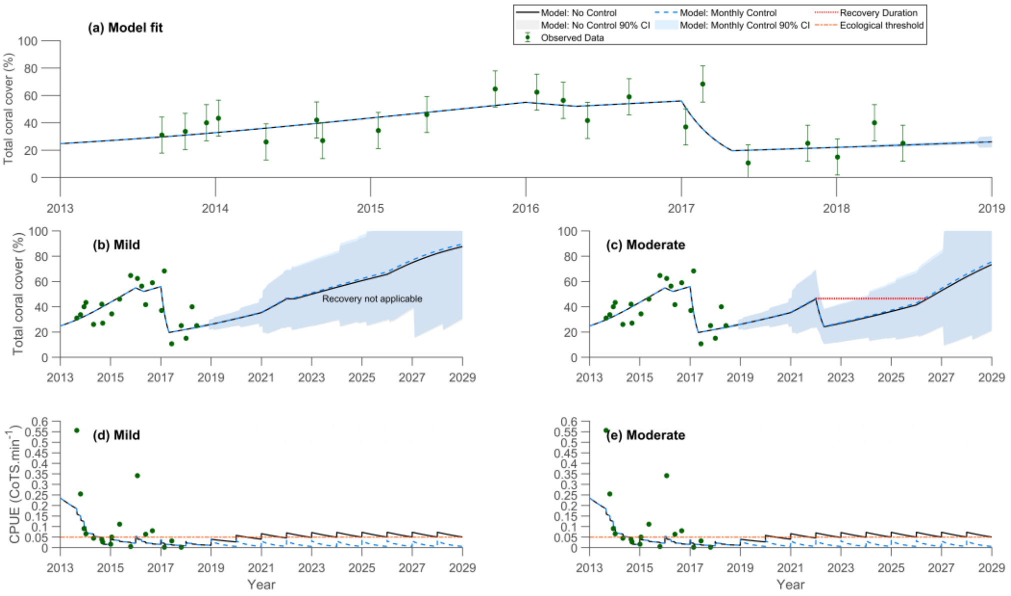

Supplementary Fig. 23: Compendium plot for Management Site 12 with details as specified in Supplementary Fig. 1 but $A = 2.5$. a The model fit for the management site, **b-c** total coral cover trajectories under different thermal stress levels expressed as Degree Heating Weeks ($DHW \in \{4, 7\}$) simulated in year 2022, and **d-e** Catch-per-unit-effort (CPUE) trajectories under $DHW = 4$ and $DHW = 7$ events.

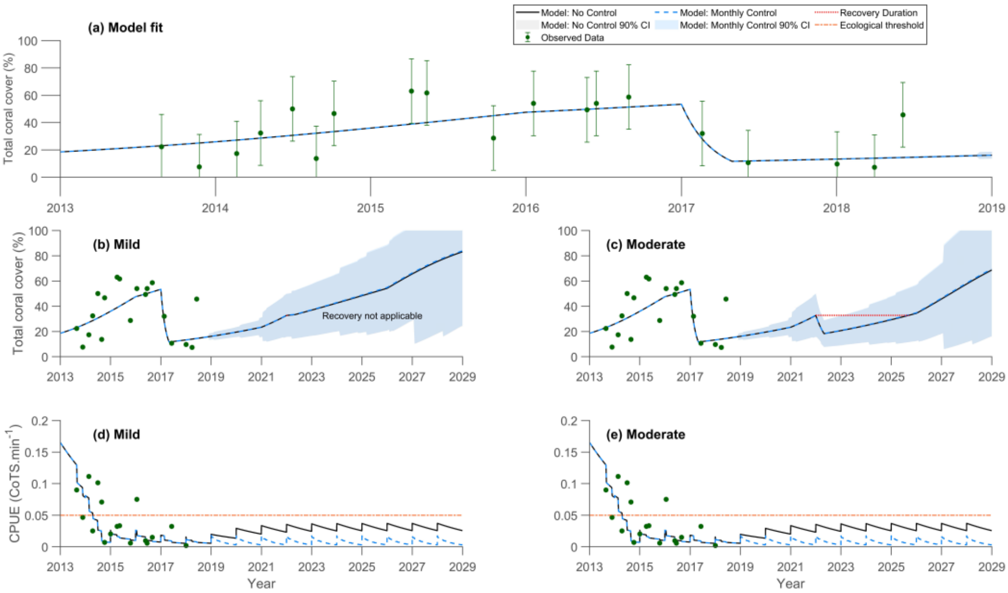

Supplementary Fig. 24: Compendium plot for Management Site 13 with details as specified in Supplementary Fig. 1 but $A = 2.5$. a The model fit for the management site, **b-c** total coral cover trajectories under different thermal stress levels expressed as Degree Heating Weeks ($DHW \in \{4, 7\}$) simulated in year 2022, and **d-e** Catch-per-unit-effort (CPUE) trajectories under $DHW = 4$ and $DHW = 7$ events.

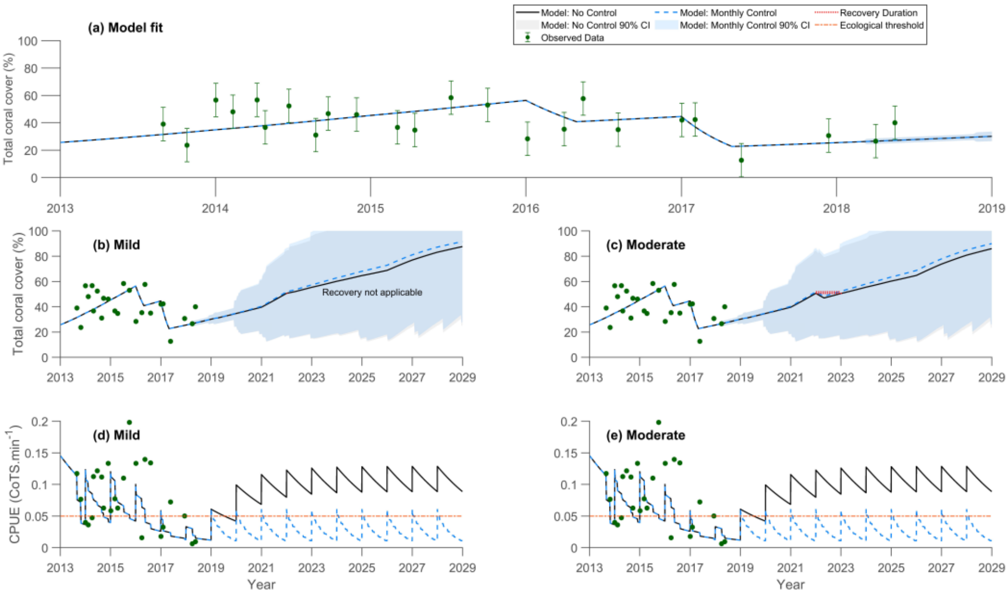

Supplementary Fig. 25: Compendium plot for Management Site 1 with details as specified in Supplementary Fig. 1 but $A = 5$. a The model fit for the management site, **b-c** total coral cover trajectories under different thermal stress levels expressed as Degree Heating Weeks ($DHW \in \{4, 7\}$) simulated in year 2022, and **d-e** Catch-per-unit-effort (CPUE) trajectories under $DHW = 4$ and $DHW = 7$ events.

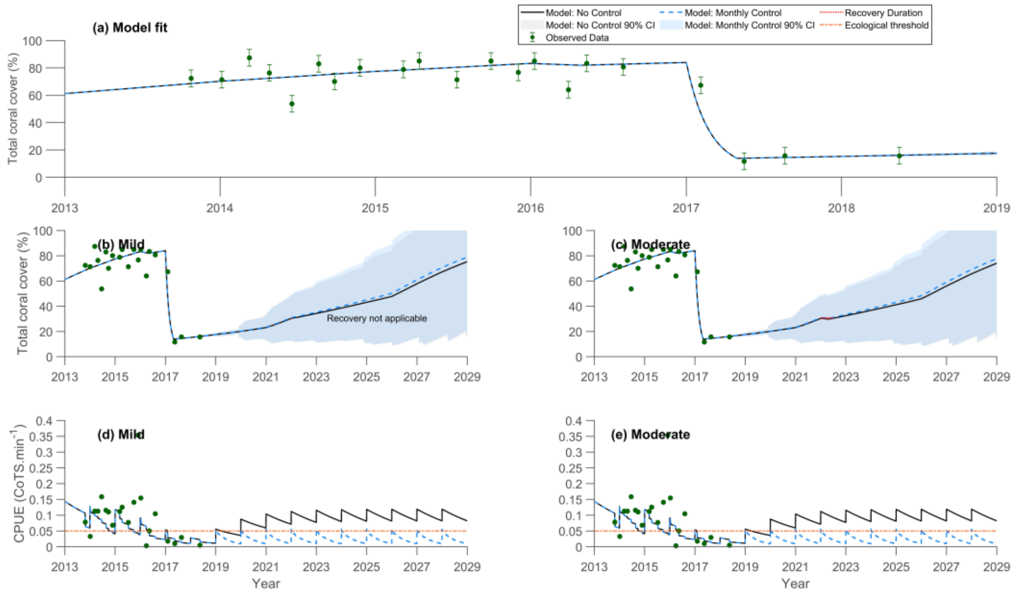

Supplementary Fig. 26: Compendium plot for Management Site 2 with details as specified in Supplementary Fig. 1 but $A = 5$. a The model fit for the management site, **b-c** total coral cover trajectories under different thermal stress levels expressed as Degree Heating Weeks ($DHW \in \{4, 7\}$) simulated in year 2022, and **d-e** Catch-per-unit-effort (CPUE) trajectories under $DHW = 4$ and $DHW = 7$ events.

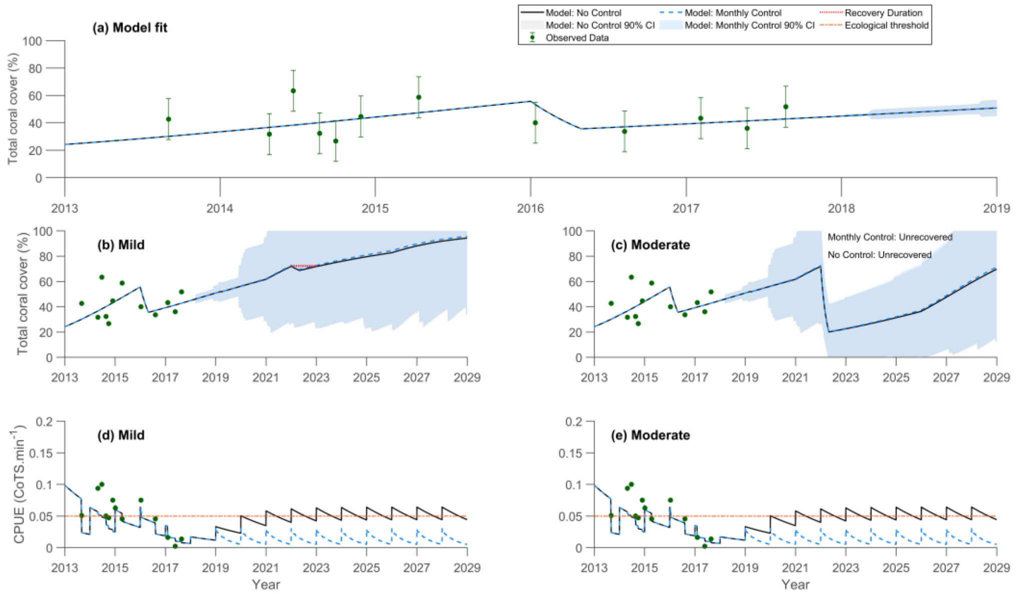

Supplementary Fig. 27: Compendium plot for Management Site 3 with details as specified in Supplementary Fig. 1 but $A = 5$. a The model fit for the management site, **b-c** total coral cover trajectories under different thermal stress levels expressed as Degree Heating Weeks ($DHW \in \{4, 7\}$) simulated in year 2022, and **d-e** Catch-per-unit-effort (CPUE) trajectories under $DHW = 4$ and $DHW = 7$ events.

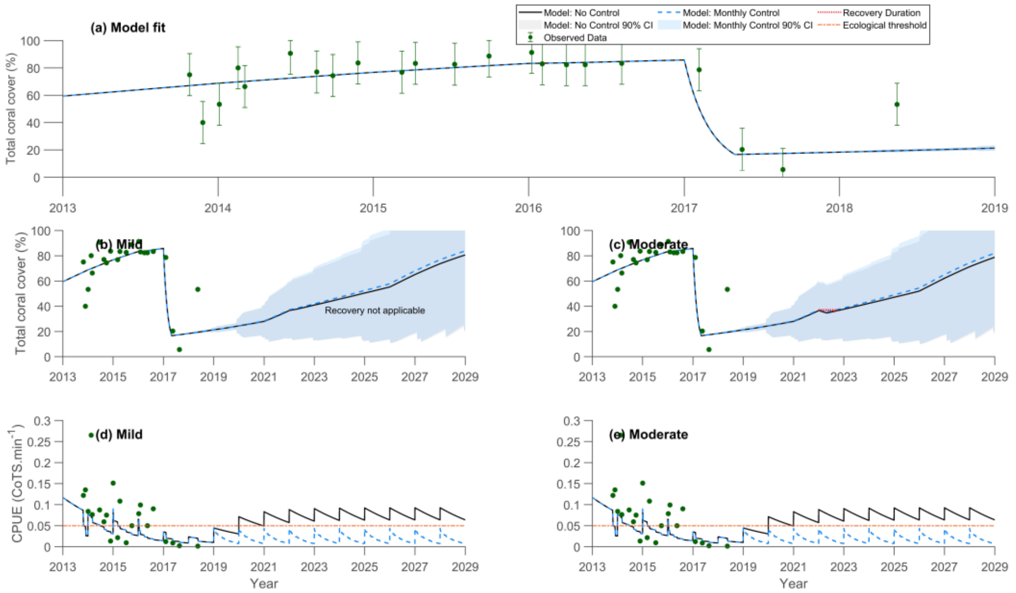

Supplementary Fig. 28: Compendium plot for Management Site 4 with details as specified in Supplementary Fig. 1 but $A = 5$. a The model fit for the management site, **b-c** total coral cover trajectories under different thermal stress levels expressed as Degree Heating Weeks ($DHW \in \{4, 7\}$) simulated in year 2022, and **d-e** catch-per-unit-effort (CPUE) trajectories under $DHW = 4$ and $DHW = 7$ events.

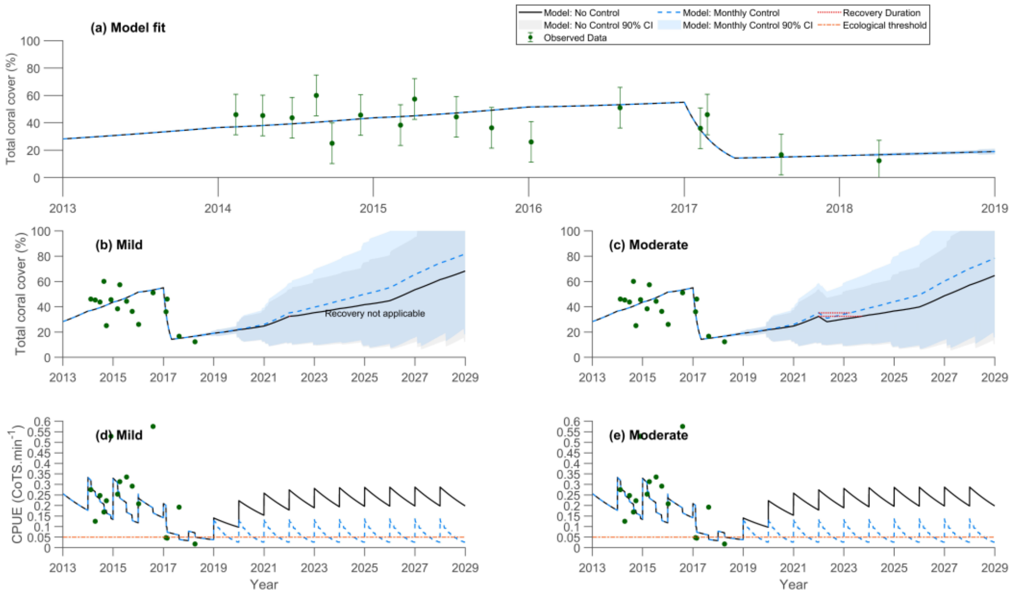

Supplementary Fig. 29: Compendium plot for Management Site 5 with details as specified in Supplementary Fig. 1 but $A = 5$. a The model fit for the management site, **b-c** total coral cover trajectories under different thermal stress levels expressed as Degree Heating Weeks ($DHW \in \{4, 7\}$) simulated in year 2022, and **d-e** catch-per-unit-effort (CPUE) trajectories under $DHW = 4$ and $DHW = 7$ events.

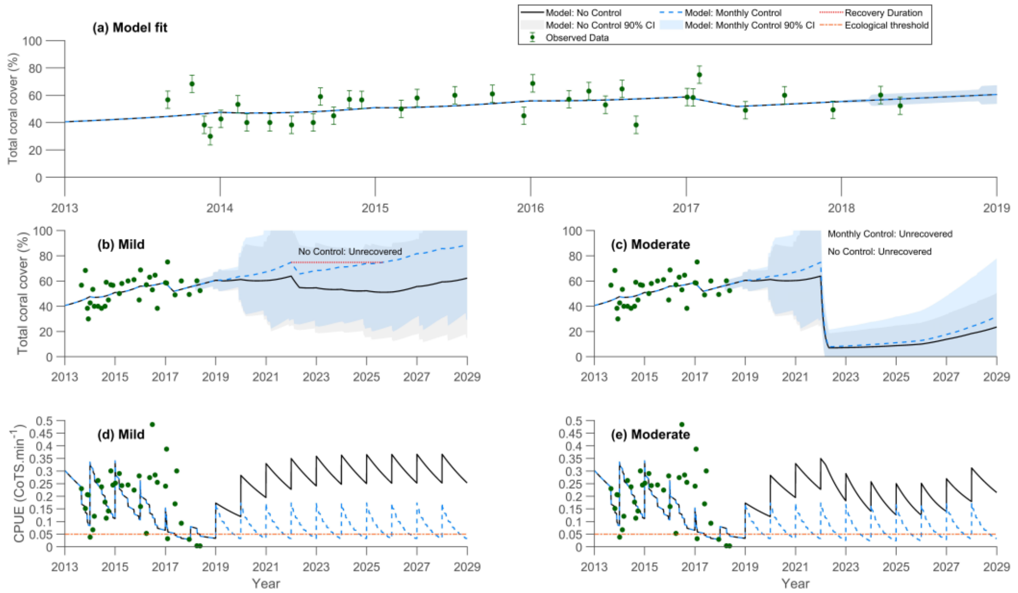

Supplementary Fig. 30: Compendium plot for Management Site 6 with details as specified in Supplementary Fig. 1 but $A = 5$. a The model fit for the management site, **b-c** total coral cover trajectories under different thermal stress levels expressed as Degree Heating Weeks ($DHW \in \{4, 7\}$) simulated in year 2022, and **d-e** catch-per-unit-effort (CPUE) trajectories under $DHW = 4$ and $DHW = 7$ events.

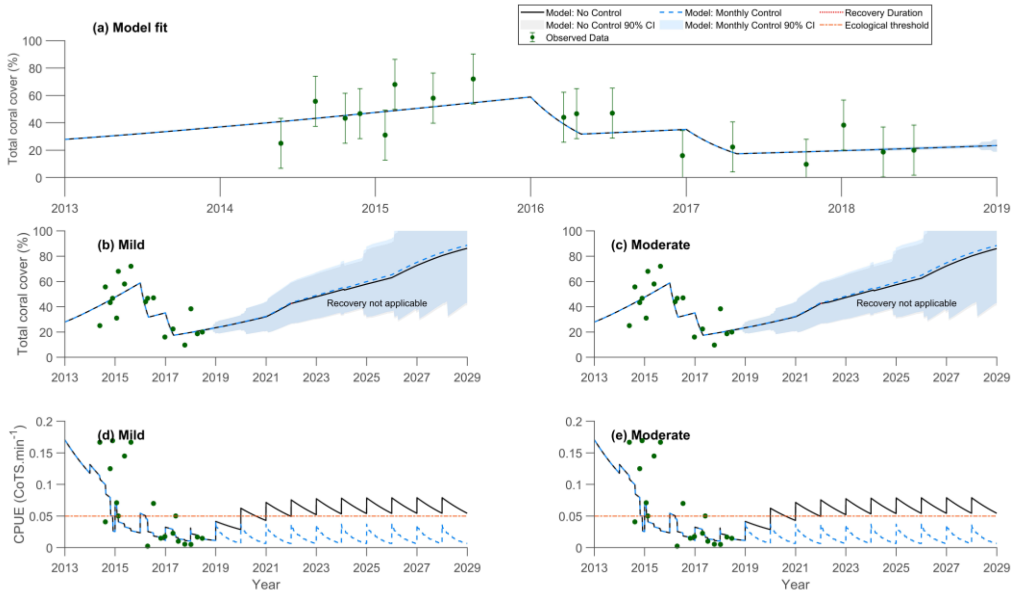

Supplementary Fig. 31: Compendium plot for Management Site 7 with details as specified in Supplementary Fig. 1 but $A = 5$. a The model fit for the management site, **b-c** total coral cover trajectories under different thermal stress levels expressed as Degree Heating Weeks ($DHW \in \{4, 7\}$) simulated in year 2022, and **d-e** catch-per-unit-effort (CPUE) trajectories under $DHW = 4$ and $DHW = 7$ events.

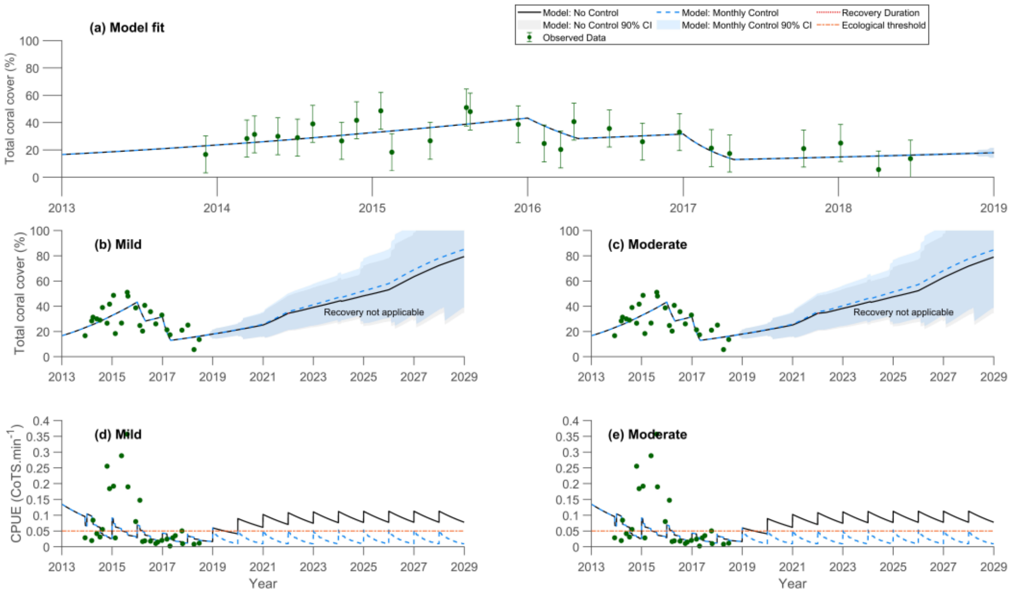

Supplementary Fig. 32: Compendium plot for Management Site 8 with details as specified in Supplementary Fig 1 but $A = 5$. a The model fit for the management site, **b-c** total coral cover trajectories under different thermal stress levels expressed as Degree Heating Weeks ($DHW \in \{4, 7\}$) simulated in year 2022, and **d-e** catch-per-unit-effort (CPUE) trajectories under $DHW = 4$ and $DHW = 7$ events.

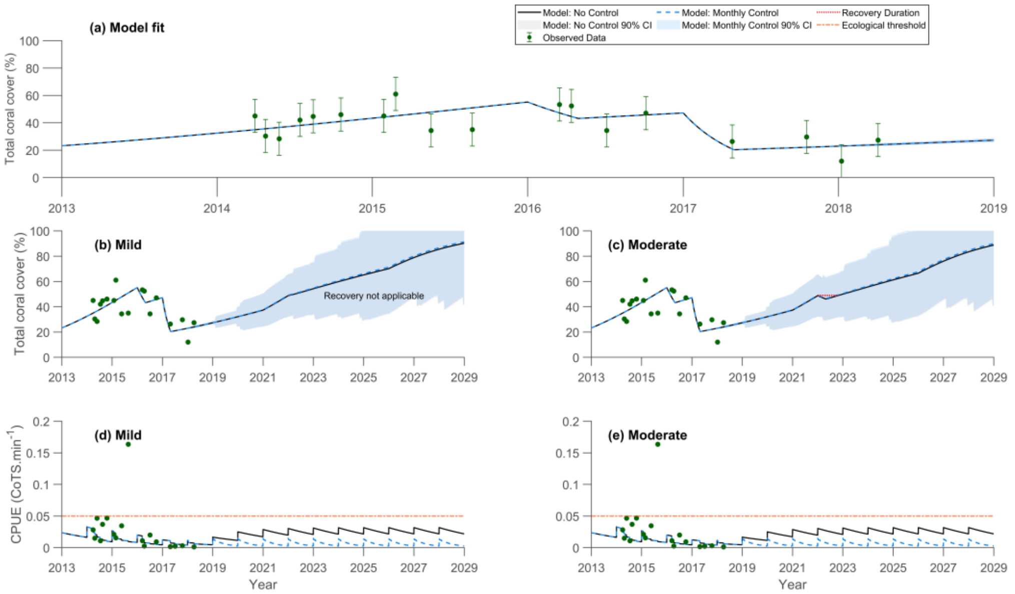

Supplementary Fig. 33: Compendium plot for Management Site 9 with details as specified in Supplementary Fig. 1 but $A = 5$. a The model fit for the management site, **b-c** total coral cover trajectories under different thermal stress levels expressed as Degree Heating Weeks ($DHW \in \{4, 7\}$) simulated in year 2022, and **d-e** catch-per-unit-effort (CPUE) trajectories under $DHW = 4$ and $DHW = 7$ events.

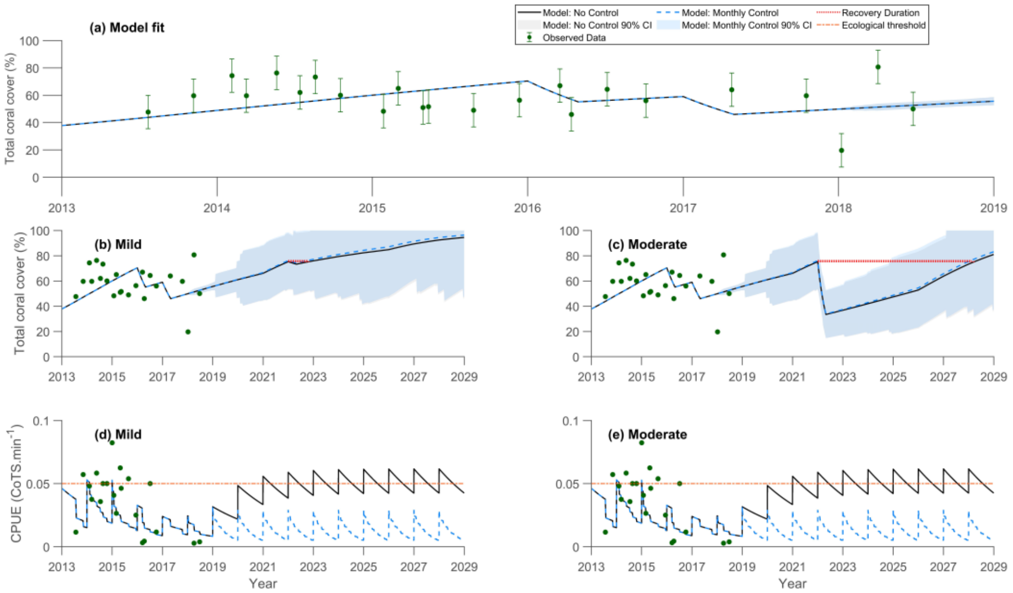

Supplementary Fig. 34: Compendium plot for Management Site 10 with details as specified in Supplementary Fig. 1 but $A = 5$. a The model fit for the management site, **b-c** total coral cover trajectories under different thermal stress levels expressed as Degree Heating Weeks ($DHW \in \{4, 7\}$) simulated in year 2022, and **d-e** catch-per-unit-effort (CPUE) trajectories under $DHW = 4$ and $DHW = 7$ events.

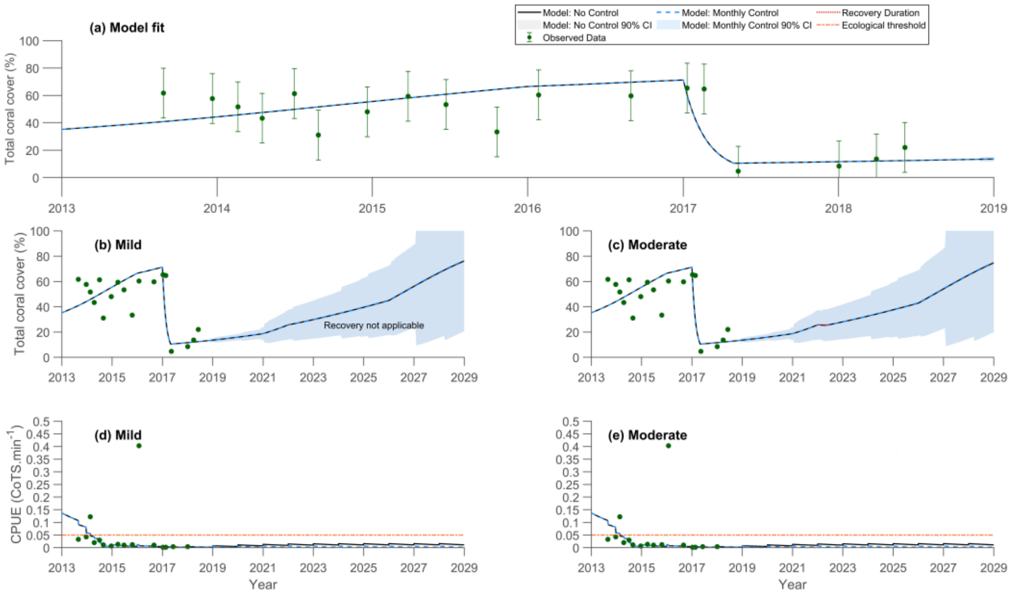

Supplementary Fig. 35: Compendium plot for Management Site 11 with details as specified in Supplementary Fig. 1 but $A = 5$. a The model fit for the management site, **b-c** total coral cover trajectories under different thermal stress levels expressed as Degree Heating Weeks ($DHW \in \{4, 7\}$) simulated in year 2022, and **d-e** catch-per-unit-effort (CPUE) trajectories under $DHW = 4$ and $DHW = 7$ events.

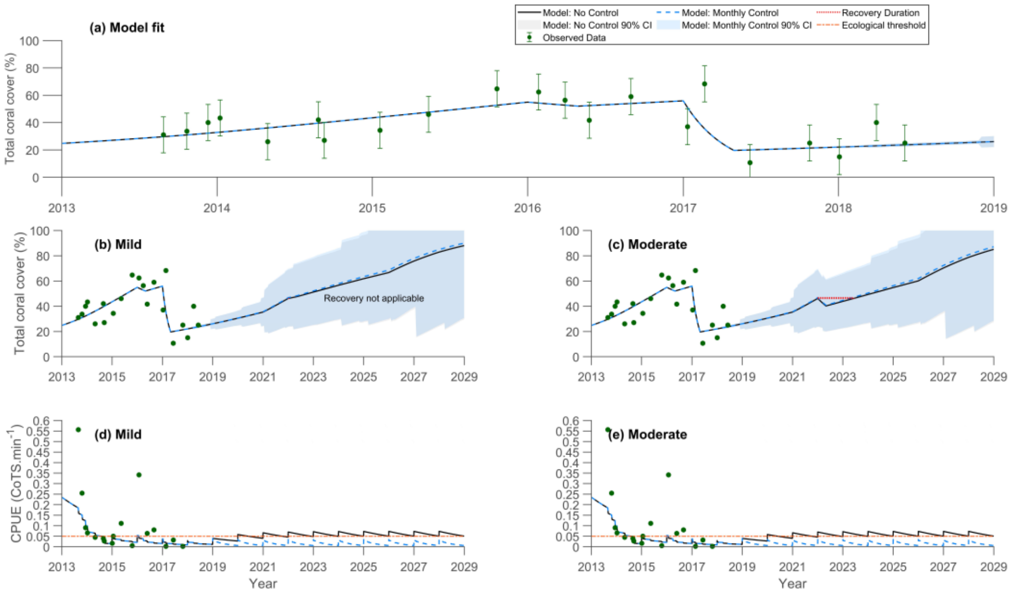

Supplementary Fig. 36: Compendium plot for Management Site 12 with details as specified in Supplementary Fig. 1 but $A = 5$. a The model fit for the management site, **b-c** total coral cover trajectories under different thermal stress levels expressed as Degree Heating Weeks ($DHW \in \{4, 7\}$) simulated in year 2022, and **d-e** catch-per-unit-effort (CPUE) trajectories under $DHW = 4$ and $DHW = 7$ events.

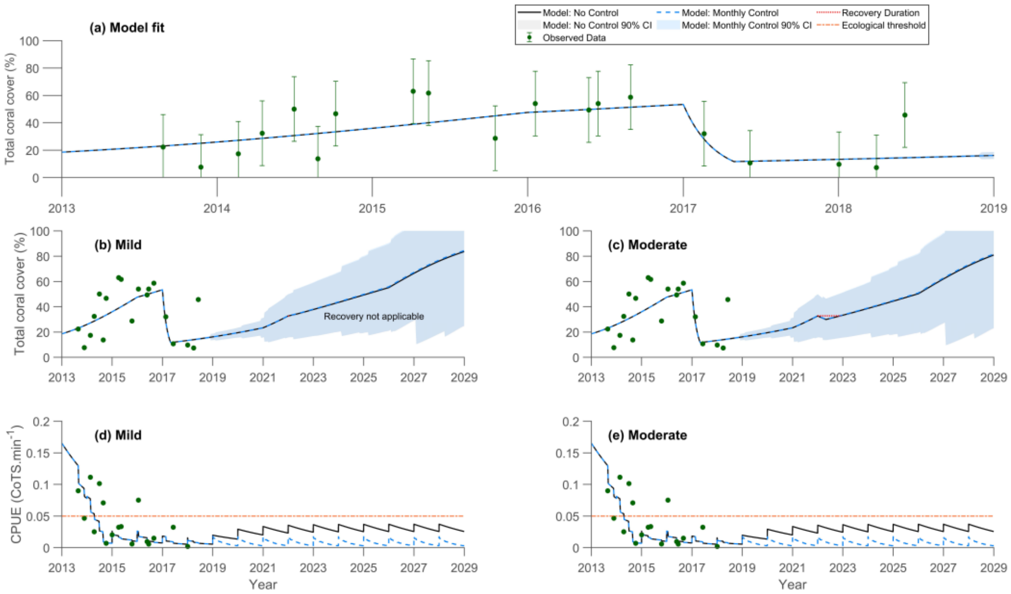

Supplementary Fig. 37: Compendium plot for Management Site 13 with details as specified in Supplementary Fig. 1 but $A = 5$. a The model fit for the management site, **b-c** total coral cover trajectories under different thermal stress levels expressed as Degree Heating Weeks ($DHW \in \{4, 7\}$) simulated in year 2022, and **d-e** catch-per-unit-effort (CPUE) trajectories under $DHW = 4$ and $DHW = 7$ events.

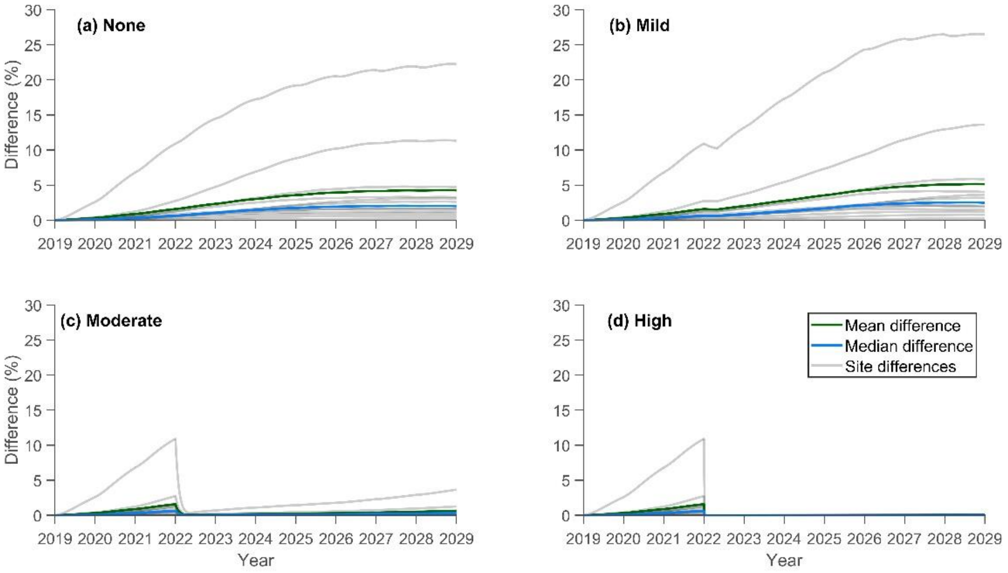

Supplementary Fig. 38: Summary of differences in coral cover between management scenarios of no manual control and monthly manual control over years 2019 t0 2029 for $\boldsymbol{A = 0}$ under different thermal stress scenarios (in terms of Degree Heating Weeks; DHW). a $DHW = 0$, **b** $DHW = 4$, **c** $DHW = 7$, and **d** $DHW = 10$. Difference represents the difference in coral cover expressed as a percentage as opposed to a proportional difference. Both mean (green line) and median (blue line) differences are plotted alongside the management-induced difference at each Management Site considered (grey lines). Differences were non-negative under all thermal stress scenarios within each Management Site.

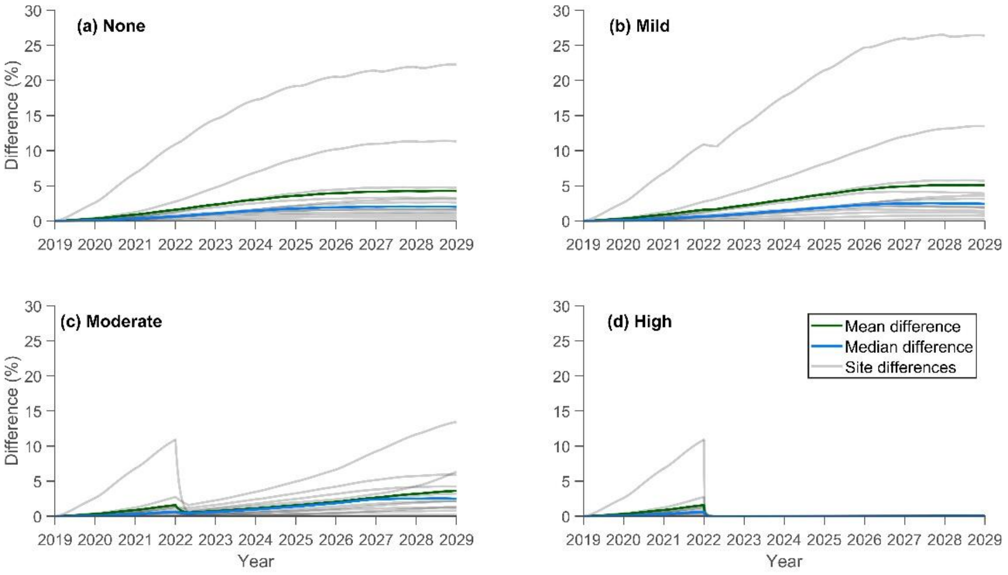

Supplementary Fig. 39: As in Supplementary Fig. 38 but for $\boldsymbol{A = 2.5}$. Summary of differences in coral cover between management scenarios of no manual control and monthly manual control over years 2019 – 2029 for $A = 2.5$ under different thermal stress scenarios (in terms of Degree Heating Weeks; DHW). **a** $DHW = 0$, **b** $DHW = 4$, **c** $DHW = 7$, and **d** $DHW = 10$.

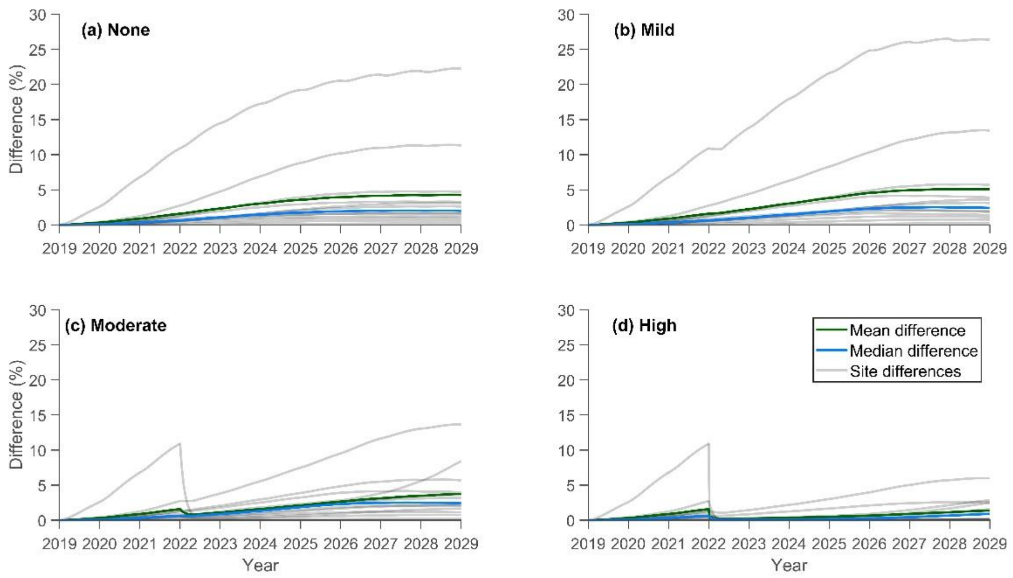

Supplementary Fig. 40: As in Supplementary Fig 38 but for $A = 5$. Summary of differences in coral cover between management scenarios of no manual control and monthly manual control over years 2019 – 2029 for $A = 5$ under different thermal stress scenarios (in terms of Degree Heating Weeks; DHW). **a** $DHW = 0$, **b** $DHW = 4$, **c** $DHW = 7$, and **d** $DHW = 10$.

-

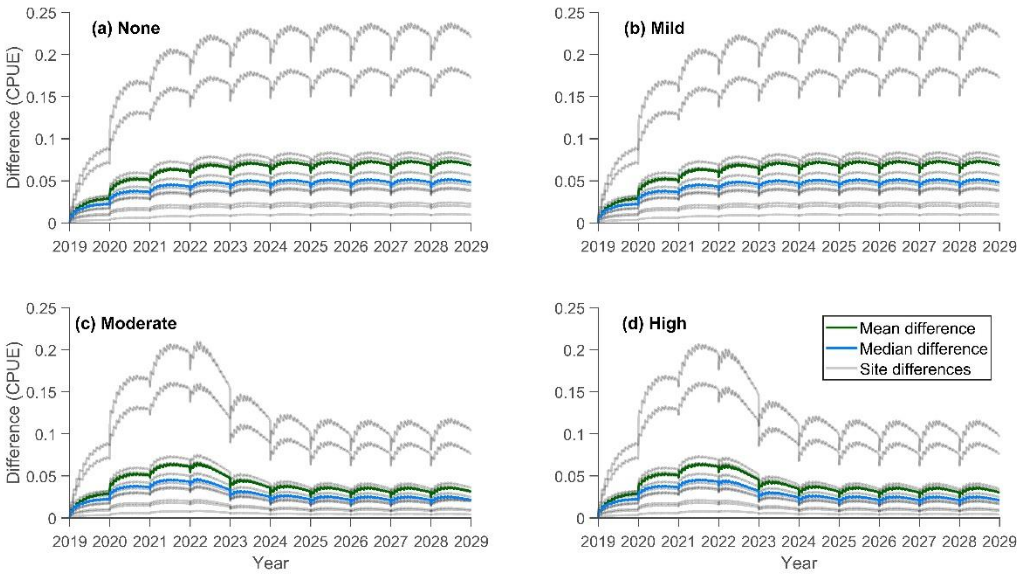

Supplementary Fig. 41: Summary of differences in Catch-per-unit-effort (CPUE; starfish.min^{-1}) between management scenarios of no manual control and monthly manual control over years 2019 – 2029 for $A = 0$ under different thermal stress scenarios (in terms of Degree Heating Weeks; DHW). a $DHW = 0$, **b** $DHW = 4$, **c** $DHW = 7$, and **d** $DHW = 10$. Difference represents the difference in CPUE. Both mean (green line) and median (grey line) differences are plotted alongside the management-induced difference at each Management Site considered (grey lines). Differences were non-negative under all thermal stress scenarios within each Management Site. Sawtooth curve patterns are due to impulses of monthly management control of CoTS whilst the concave downward periodic pattern is emergent of model population dynamics as individuals annually become detectable

to the management program in conjunction with control activities (as individuals are removed, fewer remain to be controlled and so the difference decreases after a period of increase).

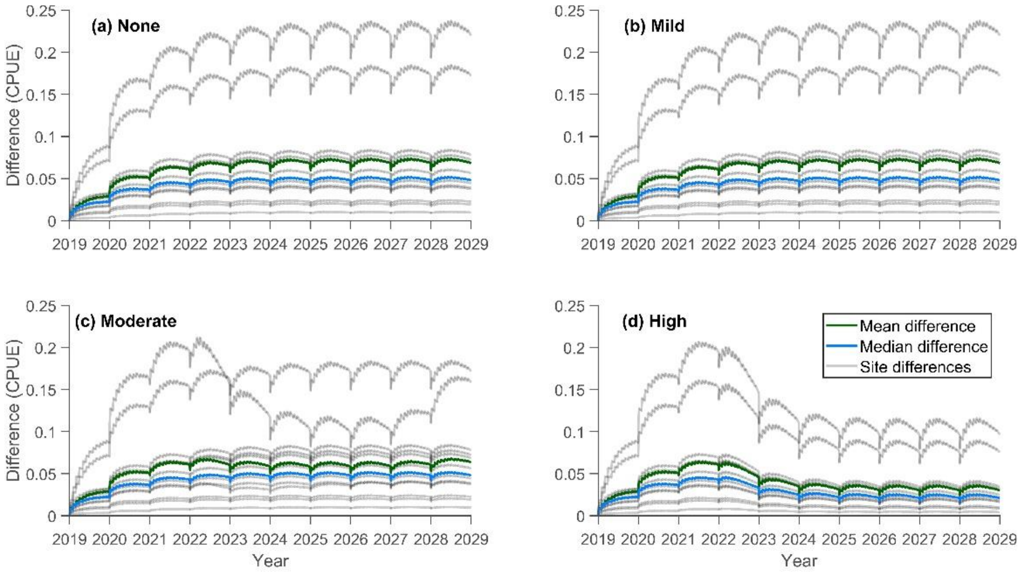

Supplementary Fig. 42: As in Supplementary Fig. 41 but for $A = 2.5$. Summary of differences in catch-per-unit-effort (CPUE; starfish.min^{-1}) between management scenarios of no manual control and monthly manual control over years 2019 – 2029 for $A = 2.5$ under different thermal stress scenarios (in terms of Degree Heating Weeks; DHW). **a** $DHW = 0$, **b** $DHW = 4$, **c** $DHW = 7$, and **d** $DHW = 10$.

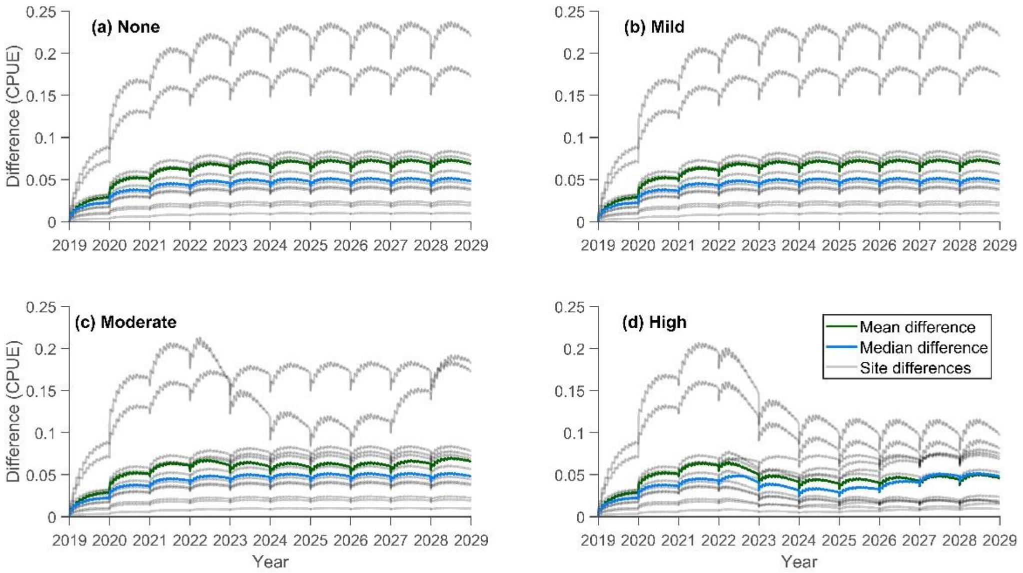

Supplementary Fig. 43: As in Supplementary Fig 41 but for $A = 5$. Summary of differences in catch-per-unit-effort (CPUE; starfish.min^{-1}) between management scenarios of no manual control and monthly manual control over years 2019 – 2029 for $A = 5$ under different thermal stress scenarios (in terms of Degree Heating Weeks; DHW). **a** $DHW = 0$, **b** $DHW = 4$, **c** $DHW = 7$, and **d** $DHW = 10$.

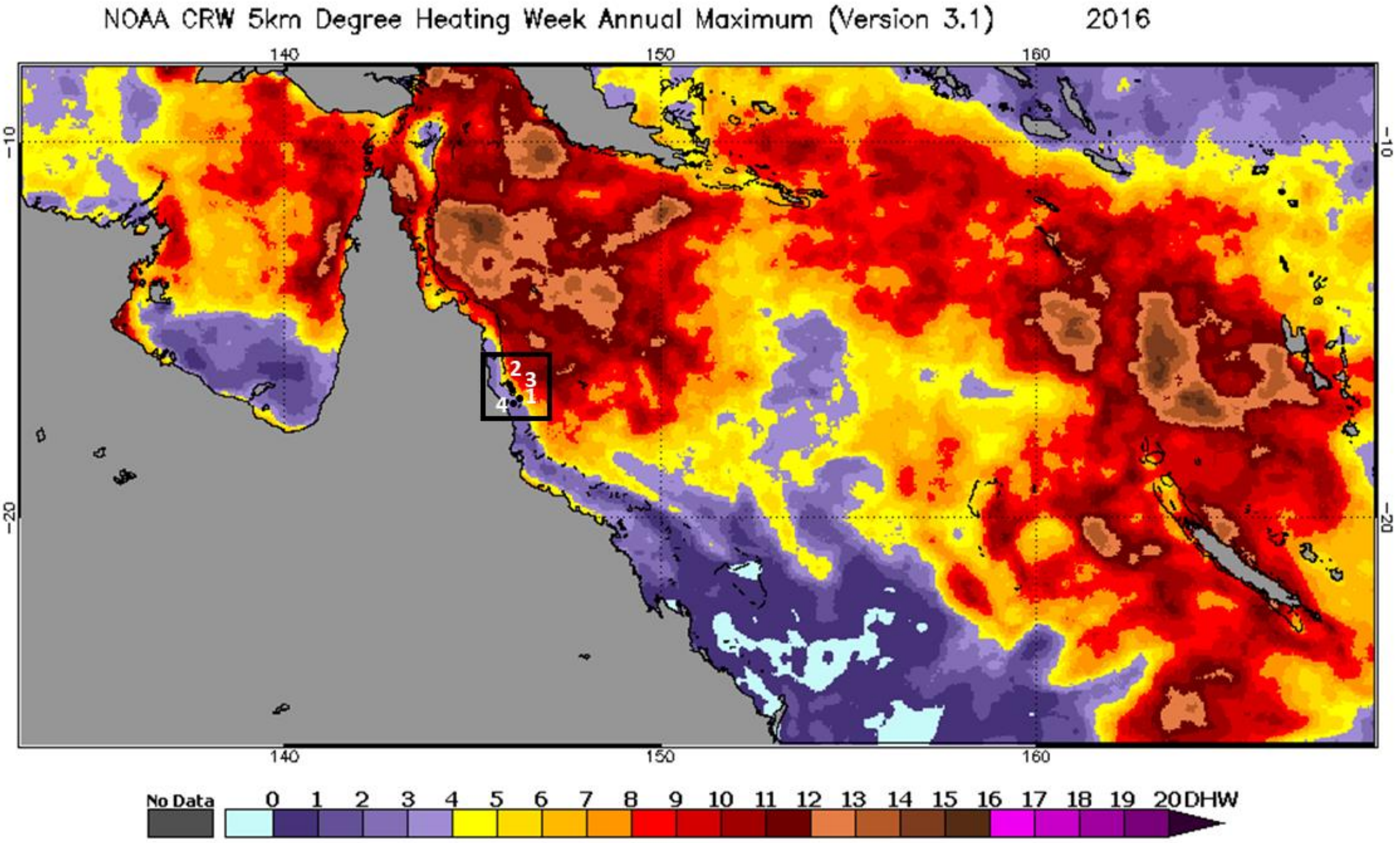

Supplementary Fig. 44: Annual composite of maximum accumulated thermal stress in terms of Degree Heating Weeks (DHW) the Great Barrier Reef over calendar year 2016. Obtained through National Oceanic and Atmospheric Administration's Coral Reef Watch [15,16] and appended with delineation of the region encompassing our four reef (black box) and approximate location of each priority reef we consider (black dots). Each reef is denoted a number (white, 1-4) that corresponds reef references throughout the rest of this study. Reef 1 experienced ~3-6 maximum DHWs, reef 2 experienced ~4-6 maximum DHWs, whilst reef 3 experienced ~4-5 maximum DHWs and reef 4 experienced a comparatively lower level of ~2-3 maximum DHWs. Discussed in supplementary text 1.

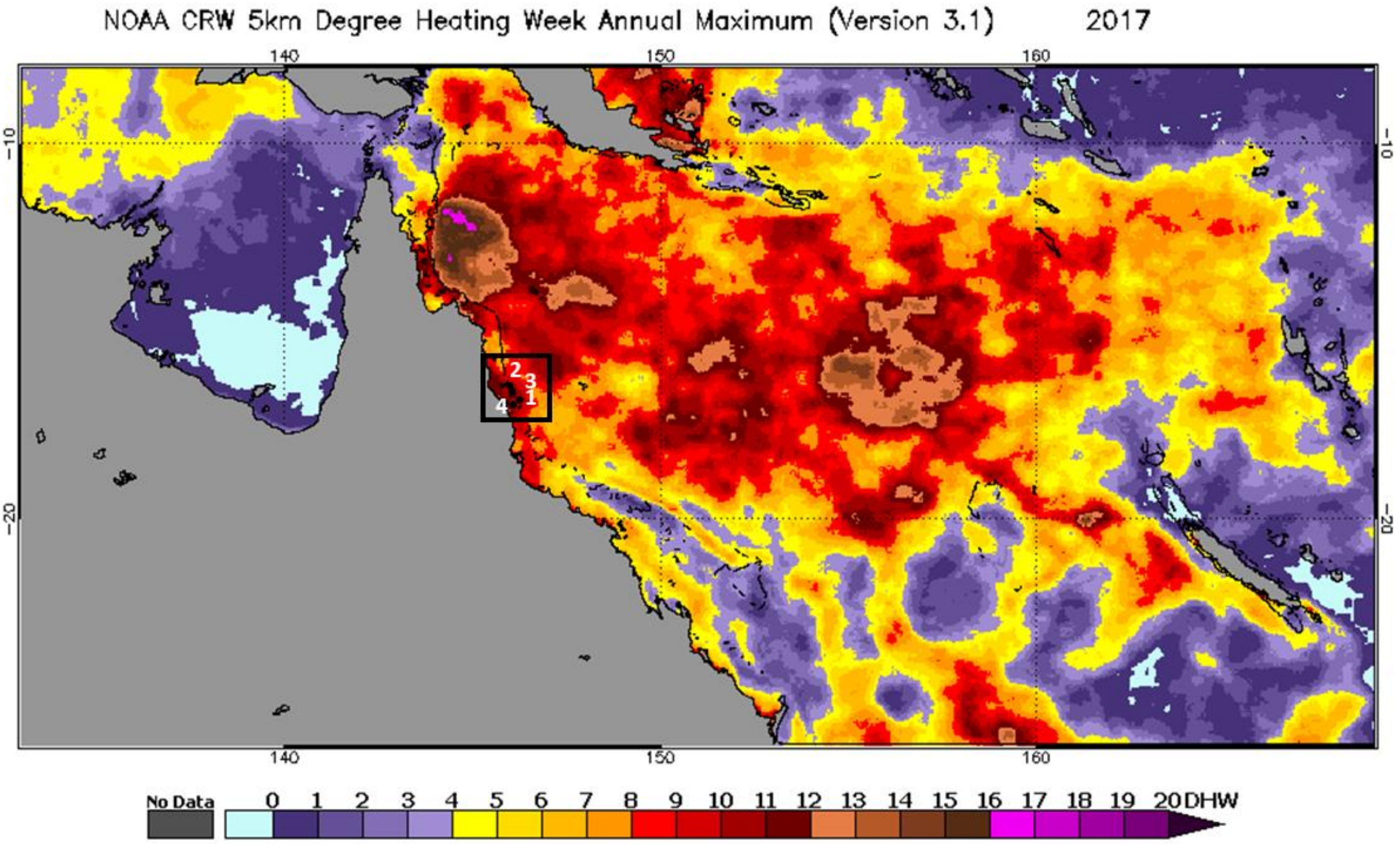

Supplementary Fig. 45: Annual composite of maximum accumulated thermal stress in terms of Degree Heating Weeks (DHW) the Great Barrier Reef over calendar year 2017. Obtained through National Oceanic and Atmospheric Administration's Coral Reef Watch[15,17] and appended with delineation of the region encompassing our four reefs (black box) and approximate location of each priority reef we consider (black dots). Each reef is denoted a number (white, 1-4) that corresponds reef references throughout the rest of this study. Reefs 1, 3, and 4 experienced ~8-9 maximum DHWs while reef 2 similarly experienced ~7-8 maximum DHWs. Discussed in supplementary text 1.

Supplementary Notes

Supplementary text 1: Validation of model fit of accumulated thermal stress

Accumulated thermal stress in terms of DHWs was estimated for each management site for both bleaching events within the central Great Barrier Reef during 2016-2017. Fitted values were subsequently validated against the National Oceanic and Atmospheric Administration (NOAA) maximum degree heating week annual composite maps [15-17] (Supplementary Fig. 44-45). The MICE model paradigm advocates the principle of using simple representations of species, processes and interactions with sufficient structure to achieve good model fits and limit complexity [18]. This approach limited model complexity otherwise required to translate accumulated thermal stress into coral population dynamics. The model-estimated maximum accumulated thermal stress at each reef was compared with NOAA estimates and deemed adequate. Model-estimated values were within ~1-2 DHWs and satisfactorily captured regional differences (Supplementary Fig. 44-45 and Supplementary Table 6). There was variability in model-estimated DHW values among management sites within each reef as would be expected based on assemblage composition, thermal history and accumulated thermal stress [19,20].

Literature cited in Supplementary Information

1 Plagányi, É. E. & Butterworth, D. S. The Scotia Sea krill fishery and its possible impacts on dependent predators: modeling localized depletion of prey. *Ecological Applications* **22**, 748-761, doi:https://doi.org/10.1890/11-0441.1 (2012).

2 Morello, E. B. *et al.* Model to manage and reduce crown-of-thorns starfish outbreaks. *Marine Ecology Progress Series* **512**, 167-183, doi:https://doi.org/10.3354/meps10858 (2014).

3 Babcock, R. *et al.* What are the important thresholds and relationships to inform the management of COTS? , (CSIRO, Australia, 2014).

4 Babcock, R., Mundy, C. & Whitehead, D. Sperm diffusion models and in situ confirmation of long-distance fertilization in the free-spawning asteroid Acanthaster planci. *The Biological Bulletin* **186**, 17-28, doi:https://doi.org/10.2307/1542033 (1994).

5 Rogers, J. G. D., Pláganyi, É. E. & Babcock, R. C. Aggregation, allee effects and critical thresholds for the management of the crown-of-thorns starfish Acanthaster planci. *Marine Ecology Progress Series* **578**, 99-114, doi:https://doi.org/10.3354/meps12252 (2017).

6 Plagányi, É. E. *et al.* Ecosystem modelling provides clues to understanding ecological tipping points. *Marine Ecology Progress Series* **512**, 99-113, doi:https://doi.org/10.3354/meps10909 (2014).

7 Plagányi, É. E., Babcock, R. C., Rogers, J., Bonin, M. & Morello, E. B. Ecological analyses to inform management targets for the culling of crown-of-thorns starfish to prevent coral decline. *Coral Reefs* **39**, 1483-1499, doi:https://doi.org/10.1007/s00338-020-01981-z (2020).

8 MacNeil, M. A. *et al.* Joint estimation of crown of thorns (Acanthaster planci) densities on the Great Barrier Reef. *PeerJ* **4**, e2310, doi:http://dx.doi.org/10.7717/peerj.2310 (2016).

9 Fabricius, K. E. *et al.* Disturbance gradients on inshore and offshore coral reefs caused by a severe tropical cyclone. *Limnology and Oceanography* **53**, 690-704, doi: https://doi.org/10.4319/lo.2008.53.2.0690 (2008).

10 Puotinen, M., Maynard, J. A., Beeden, R., Radford, B. & Williams, G. J. A robust operational model for predicting where tropical cyclone waves damage coral reefs. *Scientific Reports* **6**, 1-12, doi:https://doi.org/10.1038/srep26009 (2016).

11 Condie, S. A., Plagányi, É. E., Morello, E. B., Hock, K. & Beeden, R. Great Barrier Reef recovery through multiple interventions. *Conservation Biology* **32**, 1356-1367, doi:https://doi.org/10.1111/cobi.13161 (2018).

12 Wolff, N. H. *et al.* Temporal clustering of tropical cyclones on the Great Barrier Reef and its ecological importance. *Coral Reefs* **35**, 613-623, doi:https://doi.org/10.1007/s00338-016-1400-9 (2016).

13 Leigh, G. M., Campbell, A. B., Lunow, C. P. & O'Neill, M. F. Stock assessment of the Queensland east coast common coral trout (Plectropomus leopardus) fishery. 115 (Department of Agriculture, Fisheries and Forestry, Brisbane, 2014).

14 Westcott, D. A. *et al.* Relative efficacy of three approaches to mitigate crown-of-thorns starfish outbreaks on Australia's Great Barrier Reef. *Scientific Reports* **10**, 1-12, doi:https://doi.org/10.1038/s41598-020-69466-1 (2020).

15 Skirving, W. *et al.* Coraltemp and the coral reef watch coral bleaching heat stress product suite version 3.1. *Remote Sensing* **12**, 3856, doi:https://doi.org/10.3390/rs12233856 (2020).

16 NOAA Coral Reef Watch, 2016, *NOAA Coral Reef Watch 5km Degree Heating Week Annual Maximum (Version 3.1) 2016 for the Great Barrier Reef*, College Park, Maryland, USA, NOAA Coral Reef Watch. Accessed on 1 April 2021 at https://coralreefwatch.noaa.gov/product/5km/index_5km_composite.php.

17 NOAA Coral Reef Watch, 2017, *NOAA Coral Reef Watch 5km Degree Heating Week Annual Maximum (Version 3.1) 2017 for the Great Barrier Reef*, College Park, Maryland, USA, NOAA Coral Reef Watch. Accessed on 1 April 2021 at https://coralreefwatch.noaa.gov/product/5km/index_5km_composite.php.

18 Plagányi, É. E. *et al.* Multispecies fisheries management and conservation: tactical applications using models of intermediate complexity. *Fish and Fisheries* **15**, 1-22, doi:https://doi.org/10.1111/j.1467-2979.2012.00488.x (2014).

19 Penin, L., Adjeroud, M., Schrimm, M. & Lenihan, H. S. High spatial variability in coral bleaching around Moorea (French Polynesia): patterns across locations and water depths. *Comptes Rendus Biologies* **330**, 171-181, doi:https://doi.org/10.1016/j.crvi.2006.12.003 (2007).

20 Chou, L. M. *et al.* Differential response of coral assemblages to thermal stress underscores the complexity in predicting bleaching susceptibility. *PLOS ONE* **11**, e0159755, doi:https://doi.org/10.1371/journal.pone.0159755 (2016).
