# Supplementary material for: Culling corallivores improves short-term coral recovery under bleaching scenarios
Source: Nat Commun. 2022 May 9;13:2520. doi: 10.1038/s41467-022-30213-x (PMC9085818; doi:10.1038/s41467-022-30213-x)
Supplement: Supplementary file 3 — Description of Additional Supplementary Information [file 41467_2022_30213_MOESM3_ESM.pdf]

Supplementary Information Inventory

Culling corallivores improves short-term coral recovery under bleaching scenarios

Jacob G. D. Rogers[1, 2, *] and Éva E. Plagányi[2]

[1]School of Mathematics and Physics, University of Queensland, Brisbane 4072, Queensland, Australia.

[2]CSIRO Oceans and Atmosphere, Brisbane 4072, Queensland, Australia.

[*]Corresponding author: Jacob.Rogers@csiro.au

Item	Page
**Supplementary Table 1:** Likelihood contributions arising from each data source (coral cover data and catch-per-unit-effort data (CPUE)) and encompassed penalty terms.	2
**Supplementary Table 2:** Summary of model variables and their definitions.	4
**Supplementary Table 3:** Summary of parameters, their values and source/s where applicable.	6
**Supplementary Table 4:** Cyclone intensity, respective frequencies, wind velocities and associated damage ranges for modelled fast and slow growing coral groups on the Great Barrier Reef.	10
**Supplementary Table 5:** Summary of cyclone model parameters, values and their source/s.	11
**Supplementary Table 6:** Parameters fitted within our model across different coral thermal response scenarios alongside the spatial scale at which they were fitted.	12
**Supplementary Table 7:** Summary of data observations and effort distribution (dive minutes).	15
**Supplementary Fig. 1:** Management site 1 model fits and total coral cover (%) and catch-per-unit-effort (CPUE; CoTS.min^{-1}) trajectories under no manual control vs monthly manual control given no adaptive capacity ($A = 0$).	17
**Supplementary Fig. 2:** Compendium plot for Management Site 3 with details as specified in Supplementary Fig. 1.	18
**Supplementary Fig. 3:** Compendium plot for Management Site 4 with details as specified in Supplementary Fig. 1.	18
**Supplementary Fig. 4:** Compendium plot for Management Site 5 with details as specified in Supplementary Fig 1.	19
**Supplementary Fig. 5:** Compendium plot for Management Site 6 with details as specified in Supplementary Fig. 1.	19
**Supplementary Fig. 6:** Compendium plot for Management Site 8 with details as specified in Supplementary Fig. 1.	20
**Supplementary Fig. 7:** Compendium plot for Management Site 9 with details as specified in Supplementary Fig. 1.	20
**Supplementary Fig. 8:** Compendium plot for Management Site 10 with details as specified in Supplementary Fig. 1.	21
**Supplementary Fig. 9:** Compendium plot for Management Site 11 with details as specified in Supplementary Fig. 1.	21
**Supplementary Fig. 10:** Compendium plot for Management Site 12 with details as specified in Supplementary Fig. 1.	22
**Supplementary Fig. 11:** Compendium plot for Management Site 13 with details as specified in Supplementary Fig. 1.	22

**Supplementary Fig. 12:** Compendium plot for Management Site 1 with details as specified in Supplementary Fig. 1 but $A = 2.5$.	23
**Supplementary Fig. 13:** Compendium plot for Management Site 2 with details as specified in Supplementary Fig. 1 but $A = 2.5$.	23
**Supplementary Fig. 14:** Compendium plot for Management Site 3 with details as specified in Supplementary Fig. 1 but $A = 2.5$.	24
**Supplementary Fig. 15:** Compendium plot for Management Site 4 with details as specified in Supplementary Fig. 1 but $A = 2.5$.	24
**Supplementary Fig. 16:** Compendium plot for Management Site 5 with details as specified in Supplementary Fig. 1 but $A = 2.5$.	25
**Supplementary Fig. 17:** Compendium plot for Management Site 6 with details as specified in Supplementary Fig. 1 but $A = 2.5$.	25
**Supplementary Fig. 18:** Compendium plot for Management Site 7 with details as specified in Supplementary Fig. 1 but $A = 2.5$.	26
**Supplementary Fig. 19:** Compendium plot for Management Site 8 with details as specified in Supplementary Fig. 1 but $A = 2.5$.	26
**Supplementary Fig. 20:** Compendium plot for Management Site 9 with details as specified in Supplementary Fig. 1 but $A = 2.5$.	27
**Supplementary Fig. 21:** Compendium plot for Management Site 10 with details as specified in Supplementary Fig. 1 but $A = 2.5$.	27
**Supplementary Fig. 22:** Compendium plot for Management Site 11 with details as specified in Supplementary Fig. 1 but $A = 2.5$.	28
**Supplementary Fig. 23:** Compendium plot for Management Site 12 with details as specified in Supplementary Fig. 1 but $A = 2.5$.	28
**Supplementary Fig. 24:** Compendium plot for Management Site 13 with details as specified in Supplementary Fig. 1 but $A = 2.5$.	29
**Supplementary Fig. 25:** Compendium plot for Management Site 1 with details as specified in Supplementary Fig. 1 but $A = 5$.	29
**Supplementary Fig. 26:** Compendium plot for Management Site 2 with details as specified in Supplementary Fig. 1 but $A = 5$.	30
**Supplementary Fig. 27:** Compendium plot for Management Site 3 with details as specified in Supplementary Fig. 1 but $A = 5$.	30
**Supplementary Fig. 28:** Compendium plot for Management Site 4 with details as specified in Supplementary Fig. 1 but $A = 5$.	31
**Supplementary Fig. 29:** Compendium plot for Management Site 5 with details as specified in Supplementary Fig. 1 but $A = 5$.	31
**Supplementary Fig. 30:** Compendium plot for Management Site 6 with details as specified in Supplementary Fig. 1 but $A = 5$.	32

**Supplementary Fig. 31:** Compendium plot for Management Site 7 with details as specified in Supplementary Fig. 1 but $A = 5$.	32
**Supplementary Fig. 32:** Compendium plot for Management Site 8 with details as specified in Supplementary Fig 1 but $A = 5$.	33
**Supplementary Fig. 33:** Compendium plot for Management Site 9 with details as specified in Supplementary Fig. 1 but $A = 5$.	33
**Supplementary Fig. 34:** Compendium plot for Management Site 10 with details as specified in Supplementary Fig. 1 but $A = 5$.	34
**Supplementary Fig. 35:** Compendium plot for Management Site 11 with details as specified in Supplementary Fig. 1 but $A = 5$.	34
**Supplementary Fig. 36:** Compendium plot for Management Site 12 with details as specified in Supplementary Fig. 1 but $A = 5$.	35
**Supplementary Fig. 37:** Compendium plot for Management Site 13 with details as specified in Supplementary Fig. 1 but $A = 5$.	35
**Supplementary Fig. 38:** Summary of differences in coral cover between management scenarios of no manual control and monthly manual control over years 2019 t0 2029 for $A = 0$ under different thermal stress scenarios (in terms of Degree Heating Weeks; DHW).	36
**Supplementary Fig. 39:** As in Supplementary Fig. 38 but for $A = 2.5$.	36
**Supplementary Fig. 40:** As in Supplementary Fig 38 but for $A = 5$.	37
**Supplementary Fig. 41:** Summary of differences in Catch-per-unit-effort (CPUE; starfish.min^{-1}) between management scenarios of no manual control and monthly manual control over years 2019 – 2029 for $A = 0$ under different thermal stress scenarios (in terms of Degree Heating Weeks; DHW).	37
**Supplementary Fig. 42:** As in Supplementary Fig. 41 but for $A = 2.5$.	38
**Supplementary Fig. 43:** As in Supplementary Fig 41 but for $A = 5$.	38
**Supplementary Fig. 44:** Annual composite of maximum accumulated thermal stress in terms of Degree Heating Weeks (DHW) the Great Barrier Reef over calendar year 2016.	39
**Supplementary Fig. 45:** Annual composite of maximum accumulated thermal stress in terms of Degree Heating Weeks (DHW) the Great Barrier Reef over calendar year 2017.	40
**Supplementary text 1:** Validation of model fit of accumulated thermal stress	41
