# Supplementary material for: Culling corallivores improves short-term coral recovery under bleaching scenarios
Source: Nat Commun. 2022 May 9;13:2520. doi: 10.1038/s41467-022-30213-x (PMC9085818; doi:10.1038/s41467-022-30213-x)
Supplement: Supplementary file 4 — Reporting Summary [file 41467_2022_30213_MOESM4_ESM.pdf]

nature portfolio

Corresponding author(s): Jacob Rogers

Last updated by author(s): Apr 4, 2022

Reporting Summary

Nature Portfolio wishes to improve the reproducibility of the work that we publish. This form provides structure for consistency and transparency in reporting. For further information on Nature Portfolio policies, see our Editorial Policies and the Editorial Policy Checklist.

Statistics

For all statistical analyses, confirm that the following items are present in the figure legend, table legend, main text, or Methods section.

n/a	Confirmed	
☐	☒	The exact sample size (*n*) for each experimental group/condition, given as a discrete number and unit of measurement
☐	☒	A statement on whether measurements were taken from distinct samples or whether the same sample was measured repeatedly
☒	☐	The statistical test(s) used AND whether they are one- or two-sided<br>*Only common tests should be described solely by name; describe more complex techniques in the Methods section.*
☐	☒	A description of all covariates tested
☐	☒	A description of any assumptions or corrections, such as tests of normality and adjustment for multiple comparisons
☐	☒	A full description of the statistical parameters including central tendency (e.g. means) or other basic estimates (e.g. regression coefficient) AND variation (e.g. standard deviation) or associated estimates of uncertainty (e.g. confidence intervals)
☒	☐	For null hypothesis testing, the test statistic (e.g. *F*, *t*, *r*) with confidence intervals, effect sizes, degrees of freedom and *P* value noted<br>*Give P values as exact values whenever suitable.*
☒	☐	For Bayesian analysis, information on the choice of priors and Markov chain Monte Carlo settings
☒	☐	For hierarchical and complex designs, identification of the appropriate level for tests and full reporting of outcomes
☒	☐	Estimates of effect sizes (e.g. Cohen's *d*, Pearson's *r*), indicating how they were calculated

Our web collection on statistics for biologists contains articles on many of the points above.

Software and code

Policy information about availability of computer code

Data collection	No data collection was performed as part of this work.
Data analysis	Automatic Differentiation Model Builder (ADMB) version 12.3 was used for model fitting and simulation, MATLAB R2020b was used for visualisation, and QGIS 3.20.3 was used for study site map. For reasonable queries about the model code please email JGDR. For reasonable queries about the model code please email JGDR.

For manuscripts utilizing custom algorithms or software that are central to the research but not yet described in published literature, software must be made available to editors and reviewers. We strongly encourage code deposition in a community repository (e.g. GitHub). See the Nature Portfolio guidelines for submitting code & software for further information.

Data

Policy information about availability of data

All manuscripts must include a data availability statement. This statement should provide the following information, where applicable:

- Accession codes, unique identifiers, or web links for publicly available datasets
- A description of any restrictions on data availability
- For clinical datasets or third party data, please ensure that the statement adheres to our policy

The raw data are not available as we are not the data custodian. For reasonable request or queries about data detailing the Crown-of-thorns Starfish Control Program on the Great Barrier Reef, please email JGDR which will be redirected to the data custodian, the Great Barrier Reef Marine Park Authority. Source data (model outputs for main figures) are provided with this paper.

Field-specific reporting

Please select the one below that is the best fit for your research. If you are not sure, read the appropriate sections before making your selection.

☐ Life sciences ☐ Behavioural & social sciences ☒ Ecological, evolutionary & environmental sciences

For a reference copy of the document with all sections, see nature.com/documents/nr-reporting-summary-flat.pdf

Ecological, evolutionary & environmental sciences study design

All studies must disclose on these points even when the disclosure is negative.

Study description	We developed a novel spatial multispecies model that is also formally fitted to evaluate and quantify the role of pest corallivore culling and its influence on coral recovery trajectories. The model incorporated populations of A. cf. solaris, two groups of coral prey, coral bleaching, tropical cyclones, management culling and how these interacted with each other.
Research sample	This is a modeling study. We modeled population dynamics of A. cf. solaris and the two generalized groups of coral (nominally Acropora and Porites) on four reefs under different model parameterizations. Data the model is fitted to were obtained from the operation of the Crown-of-Thorns Starfish Control program on the central Great Barrier Reef. The present study was not involved in the program.<br><br>In the program there were fixed management sites at each reef against which data (coral cover and catch-per-unit-effort) were recorded. These management sites were modeled by the present study. There were 13 management sites across the four reefs modeled and each had coral cover and catch-per-unit-effort observations. Specifically, the sample comprised a total of 270 coral cover observations and 269 A. cf. solaris catch-per-unit-effort observations from the central Great Barrier Reef (approximately Cairns) between July 2013 and June 2018.
Sampling strategy	This is a modeling study based on observed data and did not involve experimental manipulations. The data used here were collected on reefs prioritized for control activities under operation of the Crown-of-thorns Starfish Control Program. The present study was not involved in the program but made use of the data that was collected as part of the program.<br><br>Model outcomes were averaged over 80 simulations. The number of simulations was based on hardware constraints and sufficiently large to capture variability in model simulations due to stochastic simulation of cyclone perturbations. Model components related ecological dynamics were deterministic. Cumulatively, variation was relatively low and simulations were sufficient to capture outcomes in conjunction with confidence bands.
Data collection	This is a modeling study based on observed data and did not involve experimental manipulations. Data collection was not part of this study. The model was fitted to data that were collected as part of management activities on the Great Barrier Reef which included management of A. cf. solaris and measures of coral cover at management sites. Management activities were conducted under the Crown-of-thorns Starfish Control Program. Procedures and techniques used by the control program are published here: "Great Barrier Reef Marine Park Authority 2017, Crown-of-thorns starfish control guidelines, 2nd edition, GBRMPA,Townsville". The document is available via the GBRMPA ELibrary here: https://hdl.handle.net/11017/3162. Under the program, sites were systematically swam by trained control divers with data detailing the number of starfish culled recorded post-dive. These data are recorded in an Excel spreadsheet formatted and provisioned to Crown-of-thorns Starfish Control Program operators by the Great Barrier Reef Marine Park Authority. Data recording is a mandatory requirement for operators under permit agreements. Culling operations were carried out by the Association of Marine Park Tourism Operators.
Timing and spatial scale	This is a modeling study based on observed data and did not involve experimental manipulations, this constrained the duration of and periodicity of sampling to what was available. Data were available for 2013 to 2018 within the Cairns (central) section of the Great Barrier Reef. Data were resolved to the spatial units of management sites as this was the scale at which control activities were conducted and data available. Within the data the first observation (both coral cover and catch-per-unit-effort) was 23-July-2013 and the last was 25-June-2018 (both coral and catch-per-unit-effort). The sites used in the current study were visited 20.73 ± 5.5 (mean ± 1 standard deviation) visits across the time series. Over the five years the nominal periodicity was 3.1 ± 0.8 months (mean ± 1 standard deviation).
Data exclusions	This is a modeling study based on observed data and did not involve experimental manipulations. We restricted our focus to management sites with sufficient data to avoid over parametrization of reef and management site dynamics. Each reef in the subset contained two or more management sites where each site was visited at least 18 times. The subset was used because it contained sufficient data for estimating the 11 model parameters for each management site. Using sites with fewer data did not converge or did so poorly and as such we used reefs with two or more sites using sites that had 18 or more visits.
Reproducibility	This is a modeling study based on observed data and did not involve experimental manipulations. The methodology/model is described here in detail. Modeling and simulation performed in ADMB which is freely available. A sensitivity test was performed to compare the modeled period fitted to data with the modeled projection period; results were consistent.
Randomization	This is a modeling study based on observed data and did not involve experimental manipulations. All management sites were subject to the same modeled 'treatments' (either culling or no culling, different thermal stress scenarios) and inferences based on model outputs across all sites under the 'treatment'.
Blinding	Blinding wasn't relevant here as our work is theoretical.

Did the study involve field work? ☐ Yes ☒ No

Reporting for specific materials, systems and methods

We require information from authors about some types of materials, experimental systems and methods used in many studies. Here, indicate whether each material, system or method listed is relevant to your study. If you are not sure if a list item applies to your research, read the appropriate section before selecting a response.

Materials & experimental systems

n/a	Involved in the study
☒	☐ Antibodies
☒	☐ Eukaryotic cell lines
☒	☐ Palaeontology and archaeology
☒	☐ Animals and other organisms
☒	☐ Human research participants
☒	☐ Clinical data
☒	☐ Dual use research of concern

Methods

n/a	Involved in the study
☒	☐ ChIP-seq
☒	☐ Flow cytometry
☒	☐ MRI-based neuroimaging
